# Enhanced mitochondrial function and delivery from adipose-derived stem cell spheres via the EZH2-H3K27me3-PPARγ pathway for advanced therapy

**DOI:** 10.1186/s13287-025-04164-1

**Published:** 2025-03-11

**Authors:** Ming-Min Chang, Dinh Toi Chu, Sheng-Che Lin, Jung-Shun Lee, Thuy Duong Vu, Hue Thi Vu, Thamil Selvee Ramasamy, Shau-Ping Lin, Chia-Ching Wu

**Affiliations:** 1 Department of Cell Biology and Anatomy, College of Medicine, National Cheng Kung University, No. 1, University Road, Tainan, 70101 Taiwan; 2 Medical Device Innovation Center, National Cheng Kung University, Tainan, 70101 Taiwan; 3 Faculty of Applied Sciences, International School, Vietnam National University, Hanoi, 1000 Vietnam; 4 Division of Plastic and Reconstructive Surgery, Tainan Municipal An-Nan Hospital-China Medical University, Tainan, 70965 Taiwan; 5 Division of Neurosurgery, Department of Surgery, National Cheng Kung University Hospital, Tainan, 701401 Taiwan; 6 Institute of Basic Medical Sciences, College of Medicine, National Cheng Kung University, Tainan, 70101 Taiwan; 7 Stem Cell Biology Laboratory, Department of Molecular Medicine, Faculty of Medicine, University of Malaya, Kuala Lumpur, 50603 Malaysia; 8 Institute of Biotechnology, College of Bio-Resources and Agriculture, National Taiwan University, Taipei, 10672 Taiwan; 9 Department of Biomedical Engineering, National Cheng Kung University, Tainan, 70101 Taiwan; 10 International Center for Wound Repair and Regeneration, National Cheng Kung University, Tainan, 70101 Taiwan

**Keywords:** Enhanced mitochondrial function, Adipose-derived stem cells, 3D spheroid culture, Chitosan nano-deposition, EZH2-H3K27me3-PPARγ pathway, Mitochondrial therapy

## Abstract

**Background:**

Microenvironmental alterations induce significant genetic and epigenetic changes in stem cells. Mitochondria, essential for regenerative capabilities, provide the necessary energy for stem cell function. However, the specific roles of histone modifications and mitochondrial dynamics in human adipose-derived stem cells (ASCs) during morphological transformations remain poorly understood. In this study, we aim to elucidate the mechanisms by which ASC sphere formation enhances mitochondrial function, delivery, and rescue efficiency.

**Methods:**

ASCs were cultured on chitosan nano-deposited surfaces to form 3D spheres. Mitochondrial activity and ATP production were assessed using MitoTracker staining, Seahorse XF analysis, and ATP luminescence assays. Single-cell RNA sequencing, followed by Ingenuity Pathway Analysis (IPA), was conducted to uncover key regulatory pathways, which were validated through molecular techniques. Pathway involvement was confirmed using epigenetic inhibitors or PPARγ-modulating drugs. Mitochondrial structural integrity and delivery efficiency were evaluated after isolation.

**Results:**

Chitosan-induced ASC spheres exhibited unique compact mitochondrial morphology, characterized by condensed cristae, enhanced mitochondrial activity, and increased ATP production through oxidative phosphorylation. High expressions of mitochondrial complex I genes and elevated levels of mitochondrial complex proteins were observed without an increase in reactive oxygen species (ROS). Epigenetic modification of H3K27me3 and PPARγ involvement were discovered and confirmed by inhibiting H3K27me3 with the specific EZH2 inhibitor GSK126 and by adding the PPARγ agonist Rosiglitazone (RSG). Isolated mitochondria from ASC spheres showed improved structural stability and delivery efficiency, suppressed the of inflammatory cytokines in LPS- and TNFα-induced inflamed cells, and rescued cells from damage, thereby enhancing function and promoting recovery.

**Conclusion:**

Enhancing mitochondrial ATP production via the EZH2-H3K27me3-PPARγ pathway offers an alternative strategy to conventional cell-based therapies. High-functional mitochondria and delivery efficiency show significant potential for regenerative medicine applications.

**Supplementary Information:**

The online version contains supplementary material available at 10.1186/s13287-025-04164-1.

## Background

Regenerative medicine aims to restore damaged tissues using stem cell-based interventions, with particular emphasis on adipose-derived stem cells (ASCs) due to their abundance and multilineage differentiation capacity that similar to bone marrow mesenchymal stem cells (MSCs) [1–3]. Several methods have been developed to promote sphere formation in adult stem cells, including hanging drop, gel embedding, magnetic levitation, and spinner culture [4, 5]. Multicellular systems in 3D, such as organoids, assembloids, and/or organ-on-chip models, have been vigorously developed to mimic organ functions for studying cancer, genetic disorders, infectious diseases, and neurological diseases [4, 5]. The formation of cell spheres involves self-assembly without external substrates or scaffolds. Self-assembly occurs during embryogenesis, morphogenesis, and organogenesis, and can be modulated by chemical, cellular, and microenvironmental factors [6]. Spheroid culture of ASCs or MSCs on chitosan-coated surface promotes the expression of stemness markers such as Oct4 and Nanog transcription factors and secretory factors important for cell viability, migration, and tissue regenerations [7, 8]. Chitosan, a polysaccharide derived from N-deacetylated chitin found in the exoskeletons of crustaceans and insects, possesses highly biocompatible and biodegradable properties and is widely used in biomaterials for gene delivery, cell culture, and tissue engineering. The hyaluronan-grafted chitosan surface also induced the chondrogenic markers when co-culturing ASCs with chondrocytes and assembling them into co-spheroids [9]. Additionally, chitosan-coated microenvironments can be used to sequential assembly of core-shell structures for dermal papilla spheres with an outer layer of ASCs to promote hair follicle induction [10].

Mitochondria, crucial organelles ranging from 0.5 to 1.0 μm in size, are essential for cellular energy production by oxidizing nutrients like carbohydrates, fats, and amino acids [11]. They harbor their genetic material and play pivotal roles in metabolic pathways like the tricarboxylic acid (TCA) cycle and fatty acid β-oxidation. Mitochondrial dynamics and morphology are regulated by fission, fusion, shape transition, and cytoskeletal arrangements [12, 13]. Fission involves inner membrane division and outer membrane scission, while fusion merges outer membranes followed by inner membrane joining. Shape transition controls the shift between rounded and elongated mitochondrial forms independently of fission/fusion. Mitochondrial positioning is facilitated by transport along the cytoskeleton, crucial for stable energy supply and axon maintenance in neurons. In mature cells, mitochondrial morphology undergoes dynamic changes in response to energy demands, appearing fragmented during low demand and elongated with well-developed inner membrane structures during high demand [14, 15]. Mitochondria continuously remodel through fission to divide into smaller units for mitophagy and fusion to combine into larger structures for energy production [16, 17].

Mitochondrial therapy shows promise in modulating cell metabolism, immune responses, tissue homeostasis, mitochondrial integrity, wound healing, and anti-inflammatory processes across various conditions such as acute lung injury [18], cardiac ischemic injury [19], acute myocardial infraction [20], spinal cord injury [21], stroke [22]. Dysfunction of mitochondria is notably linked to diseases or injury due to their pivotal role in energy metabolism, redox homeostasis, and signaling pathways [23]. Elevated reactive oxygen species (ROS) levels in diabetic wounds result from mitochondrial respiratory leakage and impaired cellular antioxidant defenses. Impaired mitochondria under oxidative stress release mitochondrial DNA (mtDNA), mtROS, and excessive calcium, activating the nucleotide-binding and oligomerization domain (NOD)-like receptor thermal protein domain associated protein 3 (NLRP3) inflammasomes. Mitochondrial dysfunction, leading to reduced ATP production and cell death, can be ameliorated by restoring mitochondrial content, function, and homeostasis, thus enhancing the functionality of impaired cells. Recent studies indicate the potential for mitochondria and mitochondrial DNA transfer between cells through mechanisms like tunneling nanotubes, mitochondria-derived vesicles, mitochondria-captured extracellular vesicles, and cell-free mitochondria [24]. Mitochondria can be internalized into cells without the need for transfection reagents, utilizing processes such as macropinocytosis and caveolae-dependent endocytosis [25].

Mitochondria are also recognized as key regulators of stem cell fate and function, primarily through mitochondrial metabolism and oxidative phosphorylation (OXPHOS) in the respiratory chain [26]. Increased mitochondrial biogenesis and aerobic metabolism are distinctive features of MSC differentiation, whereas mitochondrial dysfunction inhibits this process [27]. Mitochondrial networks in stem cells appear fragmented, while differentiated cells exhibit elongated tubular structures. Mitochondria enable stem cells to adapt effectively to variations in their microenvironmental conditions and play a pivotal role in metabolic regulation, acting as switches controlling stem cell differentiation and pluripotency [28]. In 2D culture, the ratio of mitochondrial area to cytoplasm increases during differentiation, suggesting the critical role of fusion/fission dynamics and mitochondrial morphology in maintaining stem cell properties [29]. The arrangement of mitochondria changes before and after MSC differentiation, with undifferentiated MSCs exhibiting perinuclear mitochondrial organization compared to the even distribution in differentiated MSCs [30]. MSC-mediated mitochondrial transfer has been investigated in various studies to exploit their therapeutic potential for repairing damaged tissues/cells [31]. Mitochondria isolated from human MSCs were nasally administered to mice, quickly reaching the meninges and being internalized by macrophages, distributing to diverse brain regions and restoring brain structure and function in cisplatin-induced cognitive deficits [32]. Local mitochondrial injection has improved regenerative muscle functions by enhancing mitochondrial dynamics and OXPHOS in muscle cells [33]. Additionally, mitochondrial transplantation through intracerebral or intraarterial injection has rescued neuronal mitochondrial dysfunction in preclinical studies of acute central nervous system injuries and neurodegenerative disorders [34]. Currently, most of mitochondria therapy involves isolated mitochondria from regular 2D MSCs or ASCs culture condition. Although stem cell behavior and function are enhanced in 3D assembly, little is known about how microenvironmental cues influence stem cell metabolic activities and their mitochondrial morphologies and functions.

Understanding the relationship between sphere formation and mitochondrial function for stem cells is an important research direction to explore the regulatory mechanisms for regenerative medicine. During ASC sphere formation, our recent single-cell RNA sequencing (scRNA-seq) study identified different cell subpopulations and epigenetic changes particularly histone modifications like histone 3 at lysine 4 (H3K4), lysine 9 (H3K9), and lysine 27 (H3K27) trimethylation are involved on ASC reprogramming and differentiation [35]. Epigenetic regulation plays a vital role in stem cell fate and functions [36], however, the detailed mechanism underlying histone trimethylation-mediated subpopulations and their mitochondrial function in ASC spheres in responses to the biomaterial microenvironments remains incompletely understood. Furthermore, while stem cell-derived compounds such as exosomes or mitochondria may play crucial roles in mediating therapeutic effects, the mechanisms governing mitochondrial internalization to rescue the damaged cells are not fully elucidated. This study aims to explore the interplay of epigenetic modifications during the assembly of ASC sphere formation and their influences on mitochondrial morphologies and functions. The contribution of chitosan nano-deposited surface and the plasticity of ASCs in the interplay of epigenetic regulation, mitochondria features, functions, isolation, and delivery are critical for enhancing the therapeutic components in stem cells and further advancing regenerative medicine.

## Materials and methods

A detailed list of chemicals and materials used in this study is provided in Supplementary Material 1, Table S1.

### Cell culture conditions

Human ASCs, MSCs harvested from normal human adipose tissue (C12977, hMSC-AT), were purchased from PromoCell GmBH and cultured in a mixture of 25% mesenchymal stem cell growth medium (C28009, PromoCell) and 75% Dulbecco’s modified Eagle’s medium high glucose (DMEM-high; 12400-061, Gibco), supplemented with 10% fetal bovine serum (FBS; Hyclone) and 1% penicillin/streptomycin (P/S) (Gibco). Hs68 cells (BCRC No. 60038), a human foreskin fibroblast cell line, were obtained from the Bioresource Collection and Research Center (BCRC) of Food Industry Research and Development Institute (FIRDI, Hsinchu, Taiwan), and the rat schwannoma cell line RT4 (RT4-D6P2T, ATCC CRL-2768) was acquired from ATCC. Both Hs68 and RT4 cells were cultured in DMEM-high media supplemented with 10% FBS and 1% P/S. All cells were maintained in a humidified incubator with 5% CO2 at 37 °C.

### 3D sphere induction

For chitosan-induced sphere formation, cells were seeded onto the chitosan-coated surface at a density of 1 × 10^6^ cells in 10 ml growth media on a 9 cm plate to induce spheroid formation for 72 h as following the established protocols [35, 37]. For sphere formation in ultra-low attachment (ULA) microplates (7007, Corning), 3000 cells were seeded per well in 200 µl growth media to assemble the 3D spheres for 72 h.

### Pathway inhibitors and treatments

The epigenetic inhibition compounds were added to the growth media at the same time as sphere induction: 70 µM SNDX-5613 (Revumenib, a KMT2A inhibitor) targeting KMT2A (MLL1) to reduce H3K4me3, 25 nM SUVi (Chateocin, a SUV39H1 inhibitor) to block H3K9me3, 10 µM GSK126 (an EZH2 inhibitor) for inhibiting H3K27me3, and 500 nM LMK235 as a HDAC5 inhibitor. The Food and Drug Administration (FDA)-approved peroxisome proliferator-activated receptor gamma (PPARγ) agonist, Rosiglitazone (RSG), was used to investigate the gain-of-function in PPARγ signaling. The PPARγ antagonist, GW9662, was applied to verify the loss-of-function in the PPARγ pathway. The RSG or GW9662 were added to the growth media during sphere formation at the concentration of 2, 5, 10, and 20 µM.

### Mitochondrial staining and observation

Prior to sphere formation on chitosan-coated surface, ASCs were incubated with MitoTracker™ Deep Red FM dye (Thermo Fisher Scientific) at a concentration of 30nM for 45 min at 37 °C. After labeling the mitochondria, cells were washed twice with PBS to remove excess dye. Following 3 days of sphere formation, the spheres were rinsed once with PBS and fixed with 4% paraformaldehyde (PFA, EM grade) in PBS at 4 °C overnight. To observe the mitochondria with cytoskeleton arrangement, rhodamine-phalloidin dye (R415, Thermo Fisher Scientific) was added and incubated overnight at 4 °C. Subsequently, the spheres were rinsed three times with PBS, permeabilized by 0.5% Triton X-100 for 2 h, and then mounted using ProLong^®^ Diamond Antifade Mountant with DAPI in the dark. Confocal microscopy was performed to examine mitochondrial morphology using the Olympus FluoView™ FV3000 confocal microscope (Olympus, Japan).

### 3D reconstruction from Z-stack images

3D image reconstruction was performed using the “Animation” function in IMARIS software (Olympus, Japan). For 2D-cultured ASCs, the Z-stack images consisted of 10 slices, while the ASC Sphere for 24 h included 87 slices (about half of the entire sphere), and the ASC Sphere for 72 h included 91 slices (about one-third of the entire sphere). The image properties were as follows: X = 0.207, Y = 0.207, and Z = 0.65.

For ASCs, the video was first rotated vertically upward by 120°, followed by a downward rotation of 40°, and then rotated counterclockwise by 45°. From an overhead view, the image was zoomed in 250x, zoomed out to the original magnification, rotated clockwise by 45°, and finally adjusted vertically downward by 80° to return to its original position. For Spheres, the process began with a vertical upward rotation of 120°, followed by a downward rotation of 30° to achieve a 90° side view. The image was then rotated counterclockwise by 150°, zoomed in 250x from an overhead perspective, and zoomed out to the original magnification. A clockwise rotation of 150° and a vertical downward rotation of 90° completed the reconstruction, returning the image to its starting position.

### Artificial intelligence-based analysis of mitochondrial morphology

For defining and quantifying MitoTracker-labeled mitochondrial shapes, the “Maximum Extent Inner” feature in the cellSens software was employed. This feature represents the maximum length of a line connecting two boundary points within the object. Subsequently, classification was performed using an artificial intelligence (AI) machine-learning-based mitochondrial neural network within Olympus cellSens Dimension software (Olympus, Japan), categorizing them into three classes based on their inner lengths: Fragmented (0.7–1.2 μm), Tubular (1.21–4.5 μm), and Compact (or Clustered) (4.51 μm and beyond). The quantification of MitoTracker fluorescent intensities in different categorized mitochondria was normalized to cell numbers by nuclei staining using DAPI or Hoechst 33342.

### Transmission electron microscopy analysis of the mitochondrial ultrastructure

Collected 3D spheres and adherent ASCs were rinsed with pre-cooled PBS and then immersed in a fixative solution containing 2.5% glutaraldehyde and 3 mM CaCl_3_ in 0.1 M cacodylate buffer for 1 h at 4 °C. The adherent 2D-cultured ASCs were then scraped into a tube. After washing with 0.1 M cacodylate buffer containing 3mM CaCl_3_, cells underwent post-fixation by immersion in 0.1 M cacodylate buffer containing 1% osmium tetroxide and 1.5% potassium ferricyanide at 4 °C for 40 min. Following the post-fixation step, the samples were washed with distilled water and then gradually dehydrated by immersion in a series of ethanol solutions with increasing concentrations: 70%, 90%, and 95%, with each stage lasting 15 min. Subsequently, the samples were immersed in 100% ethanol three times, with each immersion lasting 30 min. The dehydrated samples were then infiltrated in stages with spur resin–ethanol solutions containing 50%, then 75%, and finally 100% resin, with each stage lasting 1 h. After overnight incubation in 100% spur resin suppression, the specimens were embedded in fresh resin and polymerized at 70 °C for 24 h. The embedded samples were sectioned into 70 nm ultrathin slices using an ultramicrotome (Ultracut S, Leica Reichart) equipped with a diamond knife, and the sections were collected on nickel grids. These grids were subsequently post-stained with uranyl acetate and lead citrate. TEM analysis was performed using a JEM-1400 transmission electron microscope (JEOL, Ltd., Japan) operating at 120 keV, coupled with a 4k x 4k CCD Camera System 895 (UltraScan 4000, Gatan Inc., USA).

### Single-cell RNA sequencing assay

The scRNA-seq data from this study build upon an important aspect of our previous research. The procedures for scRNA-seq libraries preparation, sequencing, and analysis for 2D-cultured ASCs and 3D ASC spheres were detailed in a prior publication [35]. Monocle 2 software [38] was used for trajectory analysis to explore cell fate and cluster associations for investigating the cell differentiation trajectories between ASCs and chitosan-induced ASC spheres. The gene expression pattern analysis and directional predictions were conducted using Ingenuity Pathway Analysis software (IPA^®^, QIAGEN Inc, CA, USA).

### Western blotting analysis

The 2D-cultured ASCs or 3D ASC spheres were lysed using a cell lysis buffer (9083; Cell Signaling Tech) supplemented with a protease inhibitor cocktail (7012; Cell Signaling Tech) and PMSF (8553, Cell Signaling Tech). The protein concentration was measured using a Bio-Rad protein assay dye (Bio-Rad Laboratories Inc., USA). Thirty micrograms of total protein were loaded in Western blotting analysis in according to previous study [37, 39]. The target proteins were immunoblotted with specific antibodies (detailed in Supplementary Material 1, Table S2), and signal visualization was achieved using Trident femto Western HRP Substrate and a UVP EC3 imaging system (UVP Inc., Upland, CA, USA). The quantification of signal intensity was performed using ImageJ software (Image J, NIH). All observed protein levels were normalized to GAPDH and expressed as relative values, with comparisons made to the control group.

### ATP production detection assay

An ATP luminescence detection assay kit (A22066, Molecular Probes, Inc.; Invitrogen, Ltd., UK) was used to measure the ATP production of 2D-cultured ASCs and 3D ASC spheres, following the experiment procedure outlined in the manufacturer’s instructions. In brief, ten micrograms of total protein in a 10 µl cell lysate were mixed with 100 µl of assay buffer containing D-Luciferin, DTT, and firefly luciferase. The luciferase intensity was then measured at 560 nm. The ATP concentration was normalized by total protein.

### Real-time assessment of mitochondrial function using the Seahorse system

Mitochondrial oxygen consumption rates (OCR) and extracellular acidification rates (ECAR) were assessed using the Seahorse XFe24 Analyzer (Agilent, Santa Clara, CA, USA). The procedures were carried out according to the manufacturer’s instruction manual. In brief, a XFe24 cell culture microplate was coated with fibronectin (FN, 20 µg/ml) and incubated at 37 °C for 30 min. For 2D-cultured ASCs, 5000 cells were seeded onto the FN-coated plates one day before the experiment. For 3D spheroid formation, spheres were transferred and settled in microplates for an hour before the Seahorse assay.

Mitochondrial respiration was analyzed under basal conditions and in response to sequential injections of Oligomycin (1.5 µM), fluoro-carbonyl cyanide phenylhydrazone (FCCP, 1 µM), and Rotenone/Antimycin (0.5 µM each) using the Seahorse XF Cell MitoStress Test Kit. Real-time measurements for each drug were recorded over 5 cycles of 3 min in the Seahorse XF Cell Mito Stress Test program in Wave Controller 2.4 software (Agilent). The OCR was normalized to total protein content per well. The ATP production rates were determined using the Seahorse XF Real-Time ATP Rate Assay Kit following the recommended protocol. Data sets were analyzed using the Wave software and Excel software.

### RNA extraction, reverse transcriptase PCR and real-time PCR analysis

Total RNA was extracted from ASCs or spheres using TRIzol reagent (Invitrogen Corp., Carlsbad, CA, USA) and the Direct-zol RNA Miniprep Kit (Zymo Research, Inc., CA, USA), following the manufacturer’s protocol. The concentration of total RNA was determined by measuring absorbance at 260 and 280 nm using a nucleic acid spectrometer (Nanodrop ND1000, Thermo Fisher Scientific). Two micrograms of total RNA were reverse-transcribed into cDNA using the SuperScript II Reverse Transcriptase Kit (Invitrogen). The real-time PCR (qPCR) analysis was conducted using the SYBR Green Master Mix (Thermo Scientific) and ABI StepOne PlusTM (Applied Biosystems). The qPCR conditions were as follows: 50 °C for 2 min, 95 °C for 2 min, and 45 cycles of 95 °C for 3 s (s) and 60 °C for 20 s, followed by routine melting and cooling steps. The primer pairs used for qPCR in current study were listed in Supplementary Material 1, Table S3. GAPDH was employed as the reference gene for mRNA levels, and each sample was examined in triplicate. The fold change for target gene expression was calculated using the ∆∆Ct method.

### ROS assay

The cellular ROS assay kit (Ab113851, Abcam) was used to detect the occurrence of oxidative stress during 3D sphere formation. In 2D-cutlured ASCs, adherent ASCs were seeded on an 8-well chamber slide and rinsed once with PBS before being stained with 2’, 7’-dichlorofluorescin diacetate (DCFDA) for 1 h. To detect the ROS in spheres, the 3D-assembled spheres were collected in a conical tube by centrifugation and rinsed once in PBS. The spheres were stained by resuspending them in the DCFDA solution and incubating at 37 °C for 3 h. After removing the DCFDA solution, the spheres were rinsed with PBS and resuspended in complete media without phenol red, supplemented with 10% FBS. Nuclei were stained with Hoechst 33342 for 30 min before microscopy examination.

### Nuclear/Cytosol fractionation

To separate the nuclear extract from the cytoplasmic fraction of cells, the Nuclear Extraction Kit (No. 10009277, Cayman Chemical Inc., MI, USA) was used. Briefly, adherent 2D-cultured ASCs were washed with PBS containing protease inhibitors and then scraped into the hypotonic buffer. For 3D spheroids, chitosan-induced ASC spheres were transferred to a centrifuge tube and collected by centrifugation at 800 rpm for 5 min, after which the medium was discarded. Spheres were then washed with PBS containing protease inhibitors and centrifuged again before being resuspended in the hypotonic buffer. After a 15-min incubation, 200 µl of 10% NP-40 per ml of hypotonic buffer was added, and the mixture was centrifuged at 14,000 g for 30 s at 4 °C. The supernatant containing the cytosolic fraction was transferred to a new tube. The pellet was washed twice with ice-cold PBS and resuspended in the nuclear extraction buffer. To isolate the nuclear proteins, the suspension was vortexed vigorously for 15 s, incubated on ice for 15 min, followed by a 30-second vortex, and another 15-min incubation on ice. Finally, the supernatant containing the nuclear fraction was collected by centrifugation at 14,000 g for 10 min at 4 °C. Both the nuclear and cytoplasmic protein fractions were stored at -80 °C for further analyses.

### PPARγ transcription factor assay

The specific transcription factor DNA binding activity of PPARγ was detected using a PPARγ transcription factor assay kit (Ab133101, Abcam), following the manufacturer’s protocol. Briefly, ten micrograms of total protein in a 10 µl cell lysate were mixed with 90 µl Transcription Factor Binding Assay Buffer (TFB) per well of 96-well plate and incubated overnight ar 4 °C without agitation. After five PBS washes, PPARγ primary antibody was added and incubated at room temperature for 1 h. Subsequently, after five PBS washes, the secondary antibody (goat anti-rabbit HRP conjugate) was added and incubated for 1 h. To each well, 100 µl of developing solution was added and incubated for 15 min. The colorimetric absorbance was read at 450 nm within five min of adding the stop solution.

### Cell inflammation models and cell-free mitochondrial transfer experiments

#### A. Isolation of enhanced mitochondria from spheres

Mitochondria in 2D-cultured ASCs or chitosan-induced 3D spheres were extracted using Mitochondria Isolation Kit for cultured cells (Abcam, ab110170). Cells and spheres were collected by centrifugation at 800 rpm for 5 min, rinsed once with PBS, and subjected to a freeze-thaw process to disrupt the cell membrane. The samples from different groups were then suspended in Reagent A for 10 min and homogenized with 30 strokes using a Dounce homogenizer. The resultant mixture was centrifuged at 4000 rpm for 10 min. The supernatant containing Reagent A was collected. Next, Reagent B was added to resuspend the cell pellet, followed by a 10-min incubation, cell homogenization, and another centrifugation at 4000 rpm for 10 min. The combined supernatants (Reagent A and B) were centrifuged at 12,000 rpm for 15 min. The pellet was washed once with PBS, centrifuged again. After removing the PBS, the mitochondria were resuspended in growth media, and then administered to inflamed cells. Approximately 50 µg of mitochondria, quantified using the Bio-Rad protein assay, can be extracted from spheres formed by 1 × 10^6^ ASC cells in a 9 cm chitosan-coated dish after 72 h of culture. To visualized the extracted mitochondria, the MitoTracker™ Deep Red FM dye (red fluorescent color) were applied to ASCs or ASC spheres for staining and tracing the mitochondria prior isolation. The extracted mitochondria (exMito) were freshly applied for in vitro cell rescue experiments.

#### B. Inflamed cell models and treatments

For investigating the anti-inflammation and cell rescue outcome, RT4 cells (2.5 × 10^5^ cells) were seeded in 6 cm culture dishes overnight and then subjected to 20 µg/ml lipopolysaccharide (LPS) or 10 ng/ml Tumor Necrosis Factor-α (TNFα) for 3 h. The extracted mitochondria (50 µg) from various stem cell groups were added to the inflamed RT4 cells growing in a 10 cm plate and incubated for additional 48 h. The RT4 cells were harvested for Western blotting to measure the inflammatory protein expressions, and qPCR for cytokine mRNA expressions.

To observe the delivery of exMito and the intracellular mitochondrial dynamics between exMito and endogenous mitochondria, the RT4 cells were first labeled with MitoTracker™ Green FM dye (green fluorescent color) for 45 min at 37 °C before inflammatory induction by LPS or TNFα. After applying the exMito from different treatment groups for 48 h, the inflamed RT4 cells were observed using living cell confocal microscopy (Olympus FluoView™ FV3000, Olympus Corp., Japan). Fluorescent-labeled mitochondria were visualized along with the nuclei stain using Hoechst 33342 (NucBlue Live Ready Probes Reagent, Thermo Fisher Scientific) to investigate the mitochondrial morphologies and interactions between exMito (red) and endogenous mitochondria (green) within the cells.

### Statistical analysis

Statistical analysis was conducted utilizing GraphPad Prism 6 software (GraphPad, La Jolla, CA, USA). A significant threshold of p-value < 0.05 was applied to determine statistical significance in this study. Specific statistical methods corresponding to each figure were detailed in the respective Figure Legends.

## Results

### Chitosan nanodeposited surface induced 3D ASC spheres formation and mitochondria morphological chang

**Fig. 1:**
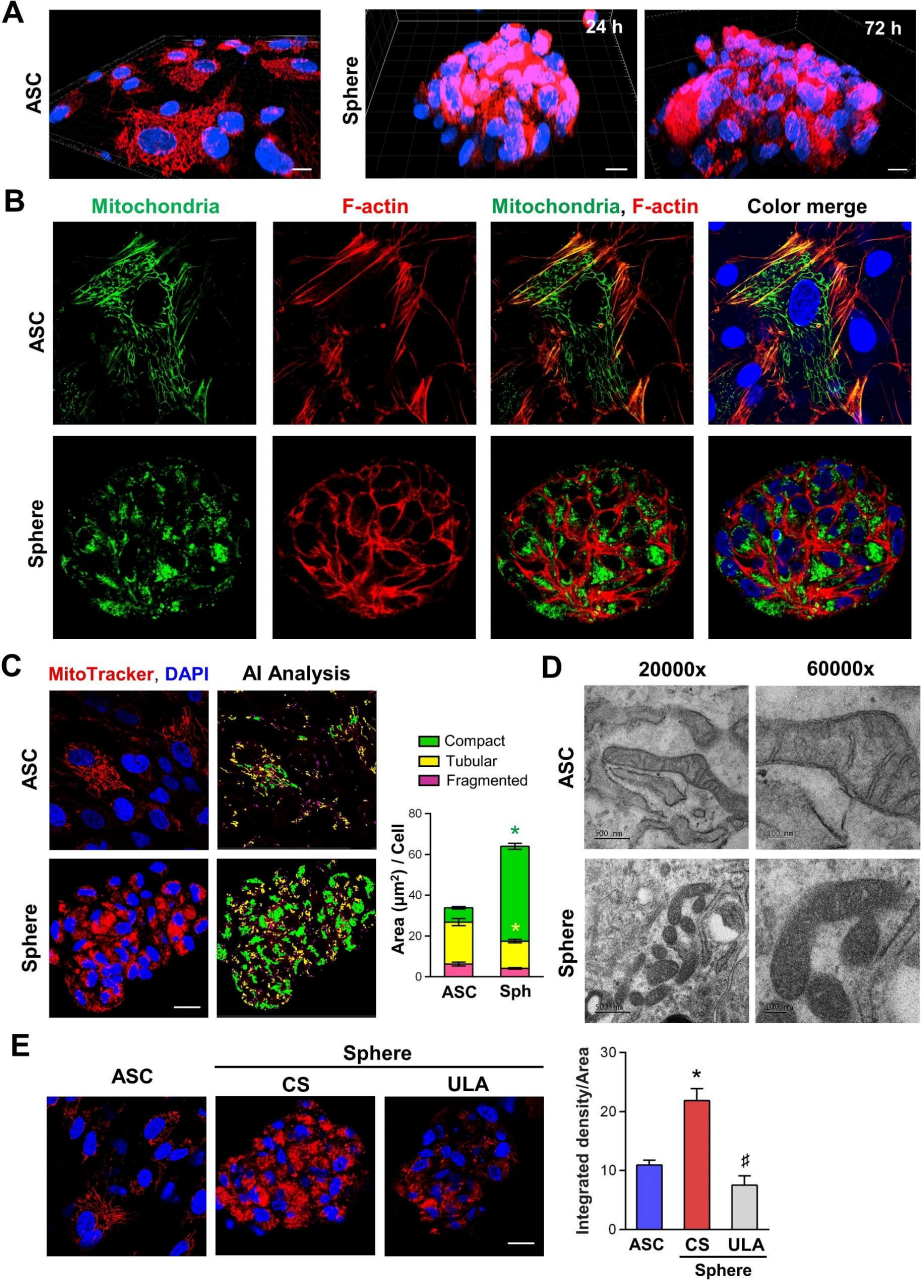
The formation of 3D spheres on chitosan nano-deposited surfaces induces morphological changes in mitochondria. (**A**) Representative screenshot images from the 3D reconstruction rotation video of mitochondria in MitoTracker Deep Red FM-stained ASCs (24 h) and chitosan-induced ASC spheres at 24 and 72 h post-induction, with DAPI staining for nuclei. (Scale bar: 15 nm). (**B**) Representative confocal microscopy images of MitoTracker Deep Red FM-labeled mitochondria and rhodamine phalloidin labeled F-actin stress fiber in ASC spheres formed by seeding ASCs on a chitosan-coated surface for 72 h. (Scale bar, 20 μm) (**C**) Mitochondrial morphologies in 2D-cultured ASCs and 3D assembled ASC spheres were identified and categorized into fragmented, tubular, and compact patterns using AI software. (Scale bar, 20 μm). The classified mitochondria were quantified by the size of each categorized area and normalized to cell number in each field of view. Values in the bar graph represent the mean ± SEM (*n* = 5) and were analyzed using Student’s *t* test, *p < 0.05. (**D**) The ultrastructure of mitochondria in ASCs and 3D spheres visualized by TEM. (Scale bar 500 nm and 100 nm for original magnification 20000x and 60000x, respectively.) (**E**) Representative mitochondrial morphologies and fluorescent intensities in ASCs and 3D spheres derived from chitosan-coated surface (CS) or ultra-low attachment (ULA) 96-well plates. (Scale bar, 20 μm). Values in the bar graph represent the mean ± SEM (*n* = 3) and were analyzed using one-way ANOVA with Tukey’s multiple comparisons post-test, *p < 0.05 compared to the ASC group. ^♯^*p* < 0.05 compared to the CS-induced sphere group

To assess mitochondrial morphology during sphere formation when seeding human ASCs on a chitosan-coated surface, we conducted mitochondrial staining with MitoTracker™ Deep Red FM dye and examined the 3D structure of mitochondria using confocal microscopy at 24, 48, and 72 h (Supplementary Material 2, Fig. S1, and Supplementary Material 3–8: Confocal Z-stack videos 1–6). Representative 3D reconstructed videos illustrated mitochondrial structure in ASC spheres at 24 and 72 h, as well as in adhered ASCs (Fig. 1A, and Supplementary Material 9–11: 3D rotation videos 1-3). In 2D-cultured ASCs, mitochondria appeared elongated, branched, and formed tubular mitochondrial networks within the cytosol. During 3D sphere formation, the mitochondria exhibited increased MitoTracker intensity with round and compact morphologies (Fig. 1A, and Supplementary Materiaal 9–11: 3D rotation videos 1–3). Additionally, the interactions between stress fibers and mitochondrial distribution were observed in ASCs and ASC spheres using double staining of F-actin and mitochondria (Fig. 1B). In ASCs, elongated mitochondria are spread out, forming a network coordinated with the cytoskeleton. In ASC spheres, F-actin was distributed on the cell surface and the mitochondria were shorter, condensed, and predominantly distributed near the cell nucleus (Fig. 1B).

To further quantify changes in mitochondrial morphology, the MitoTracker-stained mitochondria were categorized into fragmented, tubular, and compact types using Artificial Intelligence (AI)-based mitochondrial neural network software (Fig. 1C). The compact mitochondria (green color in AI analysis) showed higher expression in ASC spheres and was quantified by a significant increase in the normalized image area of compact-type mitochondria compared to tubular-type mitochondria (yellow color in AI analysis) in 2D-cultured ASCs (Fig. 1C). The Transmission Electron Microscopy (TEM) was used to further explore mitochondrial ultrastructure in ASCs in response to chitosan-induced sphere formation. Mitochondria in ASCs displayed a wide, elongated oval shape with fewer cristae and a more spaced-out arrangement (Fig. 1D). Conversely, mitochondria within 3D ASC spheres showed shorter and compacted features with compact cristae and reduced spacing.

To confirm the contribution of chitosan nano-deposition (CS) to mitochondrial induction during 3D sphere formation, the formation of ASC sphere was also assembled by seeding ASCs in an ultra-low attachment (ULA) 96-well plate. The ULA surface also promoted ASC sphere formation, but MitoTracker staining showed much less mitochondria intensity than in CS-induced ASC spheres (Fig. 1E). These results suggest the chitosan-coated surface not only benefit ASC sphere assembly but also induces mitochondria morphological changes during 3D sphere formation.

### ASC sphere formation induced specific mitochondria subpopulation

**Fig. 2:**
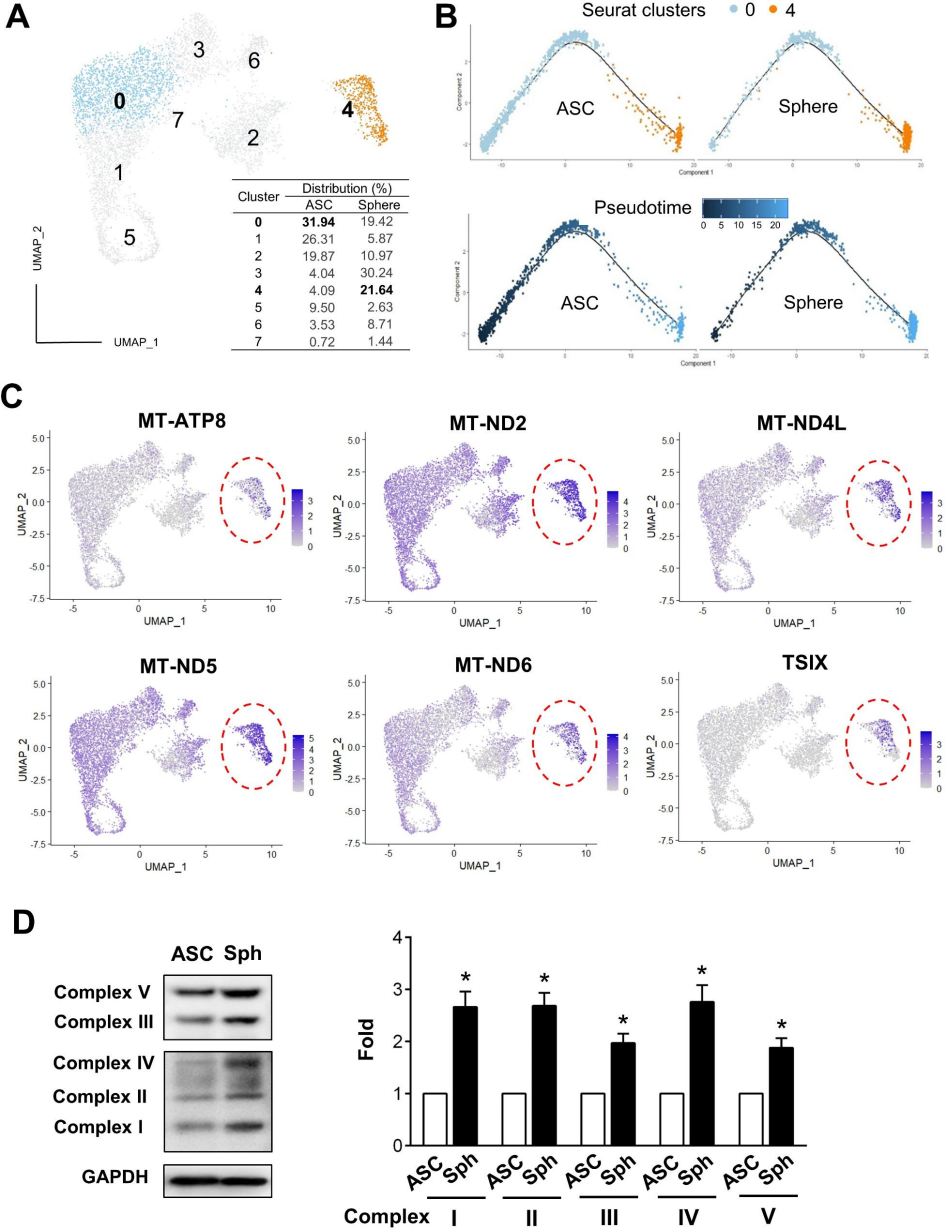
Single-cell RNA sequencing discovers a novel subpopulation in ASC spheres with heightened expressions of mitochondrial genes. (**A**) A 2D UMAP plot displays the mapping of 8 clusters within ASCs and spheres, highlighting the major subpopulations: Cluster 0 in ASCs and Cluster 4 in ASC spheres. The bottom-right panel lists the percentage distribution of each cluster. (**B**) The trajectory trees (upper panel) illustrate the distribution of Cluster 0 and Cluster 4 in ASCs and spheres. The pseudotime analysis (lower panel) shows the relationship of cell fates, where lighter blue cells indicate a more differentiated state than darker blue ones. (**C**) The highly increased genes identified in Cluster 4 cells are superimposed on the 2D UMAP plot to display their specific distribution and induction. (**D**) Representative Western blot and bar chart show the quantification of mitochondria complexes I, II, III, IV, and V levels in ASCs and 3D ASC spheres (Sph). Data were normalized to GAPDH (loading control) and represented as relative values compared to the ASC (control) group. Full-length blots are presented in Supplementary Material 12, Fig. S11. All values represent the mean ± SEM of three independent repeats (*n* = 3) and were analyzed by Student’s *t* test. *p < 0.05compared to the ASC control groupl

To further understand the genetic changes at the single-cell level during ASC sphere formation, scRNA-seq analysis was conducted to compare the 2D ASC culture and 3D ASC spheres [35]. The scRNA-seq revealed a novel cell population within ASC spheres, Cluster 4, which is characterized by its top ten genes that all associated with mitochondrial respiratory enzymes (Fig. 2A, and Supplementary Material 1, Table S4). The cell trajectory analysis illustrated that Cluster 4 in 3D spheres originated from Cluster 0 in ASCs (Fig. 2B). Among these upregulated genes, the *MT-ATP8* gene, which encodes a subunit of ATP synthase in mtDNA and crucial for OXPHOS on ATP production, is highly expressed in Cluster 4 cells (Fig. 2C, subpopulation in red circle area). The *MT-ND2*,* MT-ND4L*,* MT-ND5*, and *MT-ND6* were also highly expressed in Cluster 4 cells (Fig. 2C). These genes encode subunits of NADH dehydrogenase for mitochondria complex I to play a primary role in electrons entering the respiratory chain and serving as a rate-limiting step in overall respiration and energy production. Additionally, other mitochondrial associated genes, such as *MT-ND1*,* MT-ND3*,* MT-ND4*,* MT-ATP6*,* MT-CO1*,* MT-CO3*, and *MT-CYB*, also increased after spheroid formation (Supplementary Material 2, Fig. S2, and Supplementary Material 1, Table S4). The increases in complex I, II, and IV proteins were confirmed by western blotting analysis after ASC sphere formation (Fig. 2D).

### Enhancing mitochondrial ATP production after sphere formation and the essential roles of chitosan and ASC

**Fig. 3:**
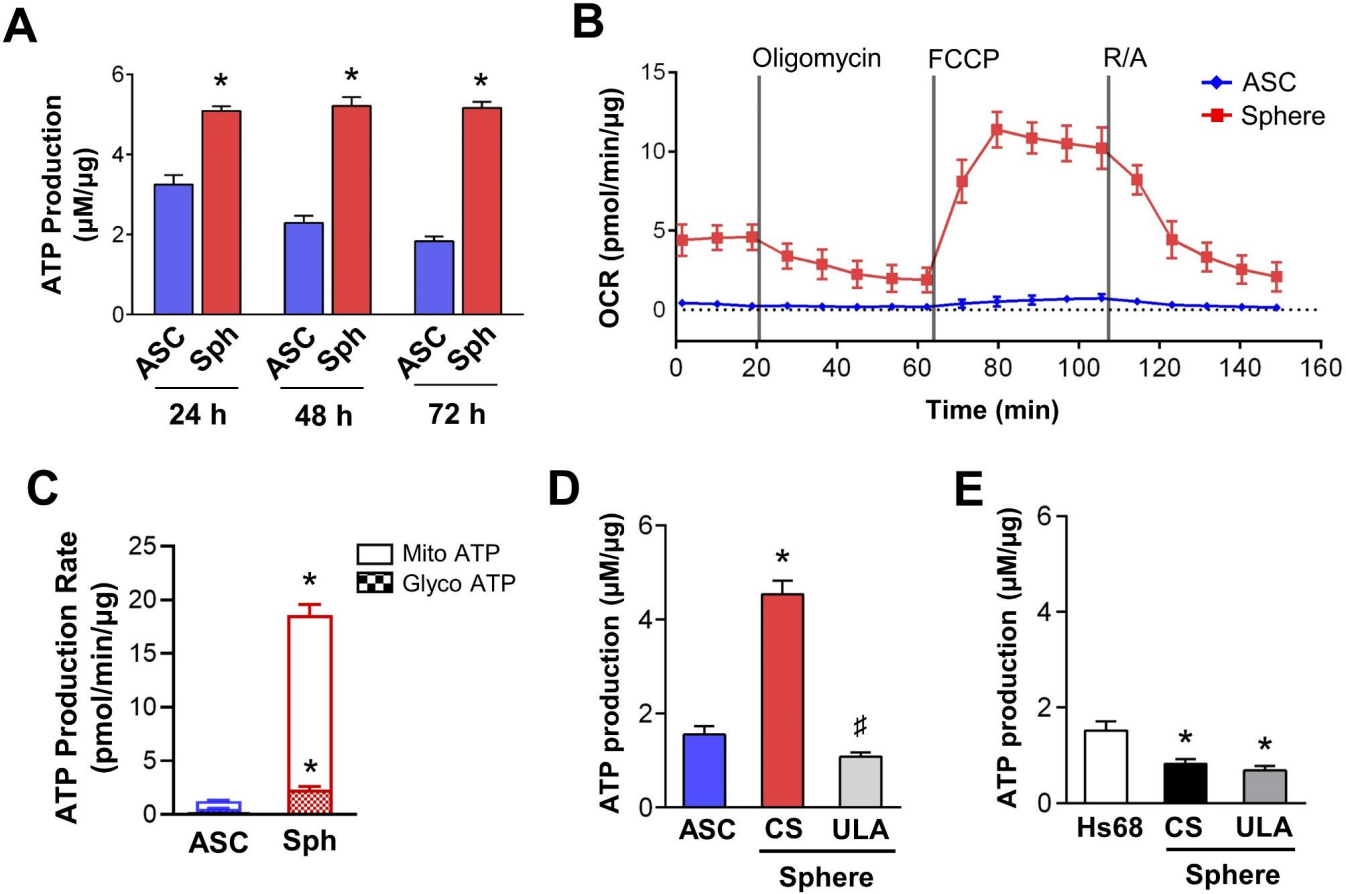
Enhanced mitochondrial ATP production was observed exclusively in chitosan-induced ASC spheres. **(A)** The ATP production, normalized to total protein levels, in ASCs and spheres was measured at 24, 48, and 72 h in 2D culture or 3D sphere formation (*n* = 4). **(B)** Seahorse real-time measurements of mitochondrial OCR in 2D-cultured ASCs and 3D assembled ASC spheres for 72 h (*n* = 3). R/A: Rotanone/Antimycin. **(C)** Seahorse metabolic flux analysis showing quantification of mitochondrial and glycolytic ATP productions (*n* = 3). **(D)** The ASC cells were seeded on chitosan-coated surfaces (CS) or ultra-low attachment (ULA) 96 well plates to form ASC spheres, and ATP production was measured 72 h after sphere induction (*n* = 4). **(E)** The ATP production was measured in Hs68 cells and CS/ULA-induced spheres (*n* = 3). All values represent the mean ± SEM and were analyzed by Student’s *t* test in (**A**) and (**C**), and one-way ANOVA with Tukey’s multiple comparisons post-test in (**D**) and (**E**). *p < 0.05 compared to the 2D-cultured ASC control group at each time point. ^♯^*p* < 0.05 compared to the CS-induced ASC sphere group

To further explore changes in mitochondrial morphology and genes, the ATP production functionality was measured using a bioluminescent ATP detection assay and revealed significant increases in ATP production after ASCs sphere formation at 24, 48, and 72 h (Fig. 3A). The Seahorse Cell Mito Stress Analysis results showed a significant increase of mitochondrial oxygen consumption rates (OCR) in spheres after fluoro-carbonyl cyanide phenylhydrazone (FCCP) injection, indicating the activation of OXPHOS in sphere mitochondria compared to 2D-cultured ASCs (Fig. 3B). The Seahorse Real-Time ATP Rate Assay demonstrated significant increases in both mitochondrial and glycolytic ATP in ASC spheres (Fig. 3C), but the increase was primarily in mitochondrial ATP after forming spheroid on chitosan-coated microenvironment. To confirm the role of chitosan in sphere-induced ATP production, we examined mitochondrial activation in a 3D sphere formation microenvironment between CS surface and ULA plates. Similar to the aforementioned mitochondria staining results, although ASCs could also form spheres in ULA plate (Supplemenraty Material 2, Fig. S3A), the ATP production did not increase compare to 2D-cultured ASCs (Fig. 3D). Additionally, we investigated whether chitosan-induced mitochondrial activation and ATP production are exclusive to ASCs. The human foreskin fibroblast (Hs68 cells) were cultured in CS or ULA microenvironments to form 3D spheres (Fig. S3B). Although Hs68 cells also formed spheres on CS surface, the ATP productions decreased in both CS and ULA Hs68 spheres (Fig. 3E). These results suggested that 3D assembly of sphere alone is not sufficient to activate mitochondria for energy production. The presence of chitosan surface and the use of ASCs are necessary factors to enhance mitochondrial ATP production in ASC spheres. Additionally, chitosan-induced sphere formation in ASCs leads to enhanced mitochondrial function, as evidenced by increased expression of genes associated with mitochondrial respiratory enzymes, suggesting a potential mechanism for improving OXPHOS energy production.

### Regulatory network and H3K27me3-EZH2 pathway for mitochondrial activations in ASC spheres

**Fig. 4:**
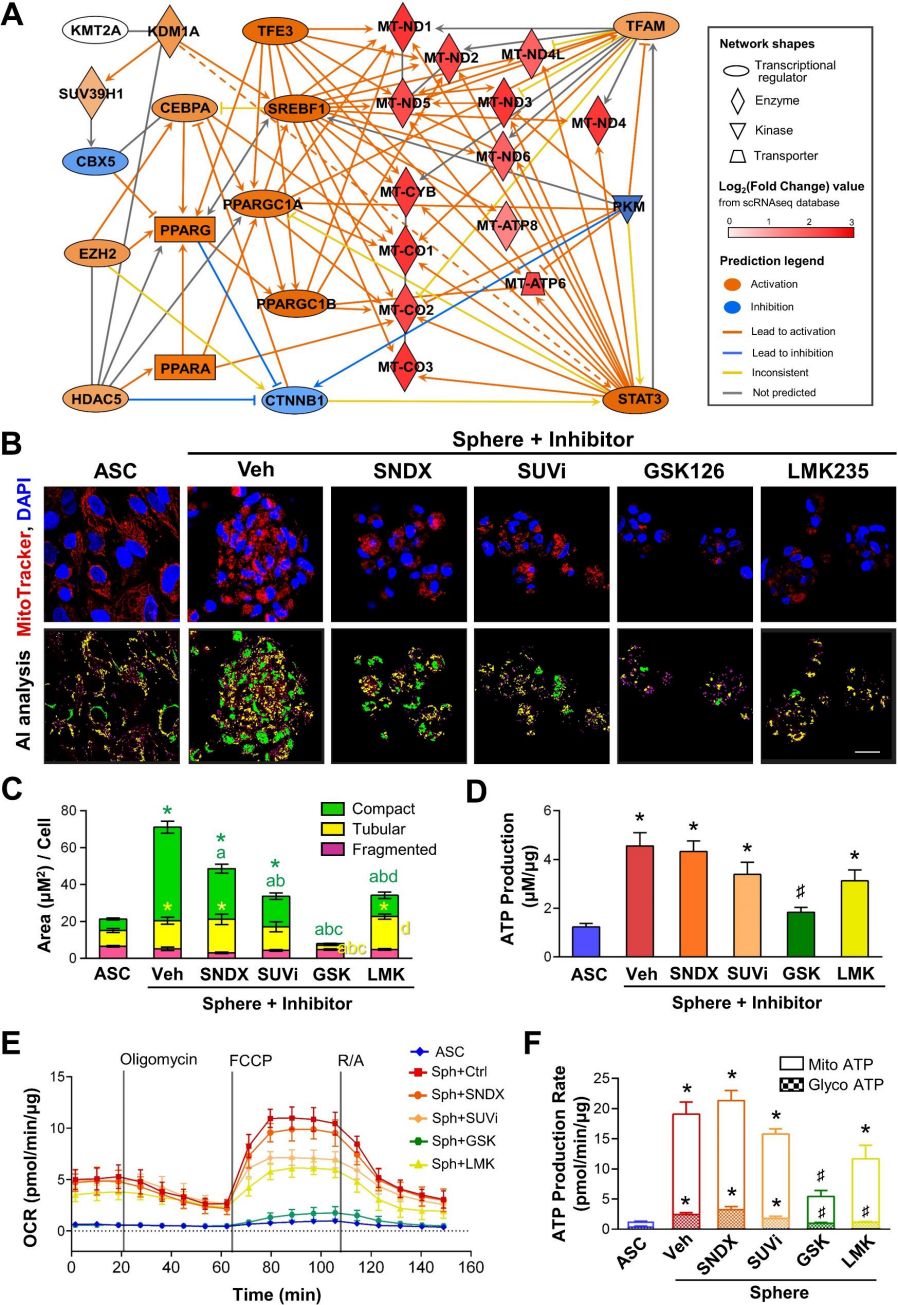
EZH2 plays a pivotal role to enhance mitochondrial functions in ASC spheres. **(A)** IPA prediction revealed the interactive network between histone H3 modification enzymes and top expressed genes in Cluster 4 cells from the scRNA-seq database. **(B)** Representative confocal images demonstrated mitochondrial morphologies, fluorescent intensities, and pattern categories in ASC spheres treated with SNDX-5613 (SNDX; 70 µM), chaetocin (SUVi; 20 nM), GSK126 (10 µM), and LMK235 (500 nM) during 72 h of sphere formation. DMSO is employed as the vehicle control (Veh). **(C)** Quantification of mitochondrial categories showed decreases in fluorescent area (µm²) per cell in GSK-treated spheres (*n* = 3). **(D)** The ATP production measured in ASC spheres with different epigenetic inhibitors described in (B) (*n* = 4). **(E)** Seahorse real-time measurements of mitochondrial OCR in ASC spheres with various inhibitor treatments (*n* = 3). **(F)** The ATP production rates for both mitochondria and glycolysis ATP in ASC spheres under various inhibitors (*n* = 3). All values represent the mean ± SEM and were analyzed by two-way ANOVA with Sidak’s multiple comparisons post-test. *p < 0.05 compared to the 2D-cultured ASC control group. ♯*p* < 0.05 compared to the CS-induced ASC sphere Veh group

To identify the potential downstream factors influenced by histone modification enzymes in mitochondrial respiration, we utilized IPA software to predict candidates by considering epigenetic regulators and the top 30 highly expressed genes in Cluster 4. The activated and inhibited effects of the signaling pathway for mitochondrial functions associate with mitochondria complex I genes and nuclear epigenetic regulation were illustrated using the Molecule Activity Predictor (MAP) overlay tool in the Ingenuity Pathway Analysis (IPA) software (Fig. 4A). Since histone trimethylation of H3K4me3, H3K9me3, and H3K27me3, and HDAC5 were previously identified in ASC spheres [35], the involvement of potential epigenetic regulators for mitochondrial functions during ASC sphere formation was examined by employing specific inhibitors of SNDX-5613, SUVi, GSK126, and LMK235 to reduce H3K4me3, H3K9me3, H3K27me3, and HDAC5, respectively. The MitoTracker Deep Red FM-labeled ASCs were seeded on chitosan-coated surface and subjected to different inhibitors for 3 days. Confocal microscopy images showed decreases in MitoTracker fluorescent intensities for the mitochondria within spheres treated with various epigenetic and HDAC5 inhibitors, particularly in GSK126-treated spheres (Fig. 4B). Spheres treated with GSK126 to inhibit H3K27me3 and EZH2 enzyme displayed predominantly punctate mitochondrial patterns and weaker fluorescence intensity. The AI quantitative analysis of mitochondria morphology also indicated that GSK126-treated spheres exhibited less compacted and filamentous mitochondria (Fig. 4C). When using the bioluminescent ATP detection assay to investigate the influence of epigenetic inhibitors on ATP production, only the GSK126 inhibitor showed a significant reduction in ATP production (Fig. 4D). Other epigenetic inhibitors, SNDX-5613, SUVi, and LMK235, did not significantly inhibit sphere-induced ATP production. The Seahorse assay further demonstrated that GSK126 treatment abolished the sphere-induced ATP production in the OCR to attenuate sphere maximal respiration and spare respiratory capacity (Fig. 4E). These results agreed with the ATP synthesis assays and real-time ATP production rate measurements that GSK126 significantly reduced total ATP synthesis, especially impacting mitochondrial ATP levels (Fig. 4F). Thus, the important roles of EZH2 and H3K27me3 in ATP production were discovered for boosting mitochondria ATP during ASC sphere formation.

### PPARγ as EZH2 key downstream pathway to boost mitochondria functions during ASC sphere fo

**Fig. 5:**
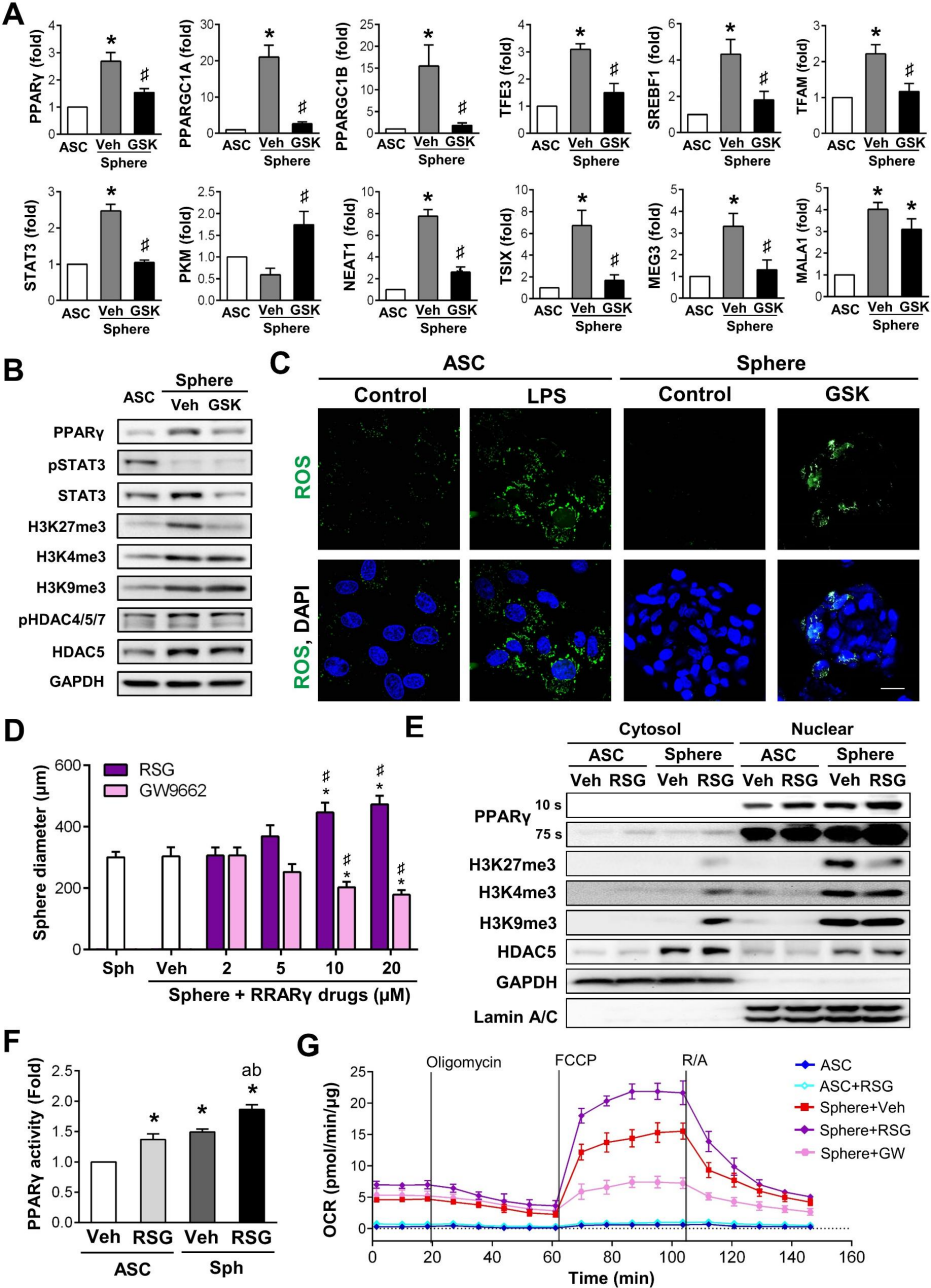
PPARγ, targeted by EZH2, is associated with mitochondrial activation in spheres. **(A)** The qPCR analysis of Cluster 4 and IPA predicted genes (PPARγ, PPARGC1A, PPARGC1B, SERBF1, STAT3, TFE3, TFAM, PKM, and Neat1) in ASCs, ASC spheres, and GSK-treated spheres. The sphere-induced PPARγ regulatory pathways and mitochondria-associated genes were abolished by GSK126 inhibitor (GSK) (*n* = 4). **(B)** Representative Western blotting of PPARγ, pSTA T3, STAT3, H3K4me3, H3K9me3, H3K27me3, p-HDAC4/5/7, and HDAC5 showed the sphere-induced protein expressions and confirmed decreases of PPARγ, STAT3, and H3K27me3 expressions by GSK inhibitor (*n* = 3). GAPDH was used as a loading control. The quantification and statistical analyses are presented in Supplementary Material 2, Fig. S4. Full-length blots are provided in Supplementary Material 12, Fig. S12. **(C)** Representative images of ROS levels in 2D-cultured ASCs without or with LPS induction (100 µM, positive control to trigger ROS), or ASC spheres with/without GSK inhibitor were observed using DCFDA staining. Nuclei were stained with Hoechst 33342. (Scale bar: 20 μm). **(D)** The sphere size increased when 10 and 20 µM of PPARγ agonist, RSG, was administered during sphere formation and was significantly reduced when the ASC spheres were treated with a PPARγ antagonist, GW9662, at 10 and 20 µM. **(E)** Representative expressions of PPARγ, H3K27me3, H3K9me3, H3K4me3, and HDAC5 in cytosol and nuclear fraction from 2D-cutlured ASCs and 3D ASC spheres with/without RSG treatments (*n* = 3). GAPDH and Lamin A/C were used as markers and loading controls for cytoplasm and nucleus, respectively. The quantification and statistical analyses are presented in Supplementary Material 2, Fig. S8. Full-length blots are presented in Supplementary Material 12, Fig. S13. **(F)** PPARγ activation was analyzed by a DNA-binding activity assay in 2D ASCs and 3D spheres with or without additional RSG **(G)** The sphere-induced mitochondrial OCR was further enhanced by treating 10 µM of PPARγ agonist RSG (Sph + RSG). Conversely, applying PPARγ antagonist GW9662 (Sph + GW) during sphere formation reduced the Seahorse real-time ATP measurements (*n* = 3). All values are represented as the mean ± SEM. Data in (**A**), (**D**), and (**F**) were analyzed by one-way ANOVA with Tukey’s multiple comparisons post-test. In (**A**) and (**D**) *p < 0.05 vs. the 2D-cultured ASC Veh group. ♯*p* < 0.05 vs. the CS-induced ASC sphere Veh group. In (**F**), *p < 0.05 vs. the 2D-cultured ASC Veh group. ap < 0.05 vs. the ASC-RSG group. bp < 0.05 vs. the CS-induced ASC sphere Veh group

According to IPA predictions, the EZH2 enzyme directly targets to PPARγ (also known as PPARG) and CEBPA for regulating a wide range of mitochondrial proteins. The *PPARG*, *PPARG coactivator 1 alpha (PPARGC1A)*,* PPARG coactivator 1 beta (PPARGC1B)*,* sterol regulatory element-binding protein 1 (SREBF1)*, and *transcription factor for Immunoglobulin Heavy-Chain Enhancer 3 (TEF3)* were suggested to be the potential signals influencing mitochondrial genes. Other genes, like *pyruvate kinase M1/2 (PKM)*, *signal transducer and activator of transcription 3 (STAT3)*, and *mitochondrial transcription factor A (TFAM)*, were also identified for mitochondrial functions. The increases of mRNA levels in *PPARγ*,* PPARGC1A*,* PPARGC1B*,* SREBF1*,* TFAM*,* TEF3*, and *STAT3* genes, as conformed by qPCR analysis, indicate the activation of PPARγ and its associated pathways during sphere formation (Fig. 5A). Additionally, these ASC sphere-induced genes were significantly inhibited by GSK126 treatment. The glycolytic enzyme PKM is responsible for converting phosphoenolpyruvate into pyruvate [40]. The decrease of PKM mRNA levels in spheres also suggests that ATP production during sphere formation may predominantly occur through mitochondrial ATP generation. Furthermore, the application of GSK126 in ASC spheres abolished mitochondrial ATP and increased the PKM gene, which may switch to glycolytic ATP (Fig. 5A). The involvement of PPARγ pathways was verified by Western blotting during sphere formation with or without the EZH2 inhibitor GSK126 (Fig. 5B, and Supplementary Material 2, Fig. S4). The inhibition of the EZH2 enzyme during sphere formation not only blocked H3K27me3 but also inhibited the sphere-induced expressions of PPARγ and STAT3. Other epigenetic and HDAC5 inhibitors including SNDX, SUVi, and LMK235, were also investigated for their effects on blocking H3K4me3, H3K9me3, and HDAC5, as well as on the aforementioned mRNA (Supplementary Material 2, Fig. S5) and protein (Supplementary Material 2, Fig. S6) expressions. Some mitochondrial target genes were inhibited by the SUVi inhibitor, such as *PPARγ*, *PPARGC1B*, *TFE3*, *SREBF1*, and *STAT3*, but these inhibitions were not as broad as that observed with GSK126.

Mitochondria generate O2•− for electron transport mainly in complex I and complex III. However, a small fraction of oxygen escapes from the mitochondria during the process of ATP production may contribute to ROS formation. To further investigate whether the increases of mitochondrial complex I in ASC spheres will induce ROS levels, we used DCFDA microscopic staining to observe the cellular ROS between 2D-cutlured ASC and chitosan-coated 3D ASC spheres. Similar to other studies showing low ROS levels in ASCs [41], the ROS expression was almost undetectable in 2D spread-out ASCs and could be triggered by the common inflammation inducer, lipopolysaccharide (LPS) (Fig. 5C, ASC groups). Although complex I genes and proteins increased in ASC spheres, the ROS levels remained low (Fig. 5C, sphere control). Inhibition of the EZH2 enzyme by GSK126 during ASC sphere formation increased ROS production (Fig. 5C, sphere with GSK). These findings suggest that mitochondrial complex I in ASC spheres is independent of ROS production when seeding ASCs on a chitosan-coated surface. Moreover, blocking the EZH2 enzyme during sphere formation increased ROS, further highlighting the important role of H3K27me3 in high functional mitochondrial production in ASC spheres.

To ascertain the role of PPARγ in the mitochondrial function of ASC spheres, the PPARγ agonist RSG that commonly used for type II diabetes mellitus treatment was applied to confirm the gain-of-function in PPARγ regulatory mechanism during sphere formation. The PPARγ antagonist GW9662 was also used to test the lost-of-function in PPARγ pathway. Treatment with both RSG and GW9662 during sphere formation resulted in dose-dependent effects on sphere size (Fig. 5D, phase images for sphere morphologies in Supplementary Material 2, Fig. S7A). Sphere size was significantly increased in response to the 10 and 20 µM RSG treatment, while it significantly decreased with 10 and 20 µM GW9662 treatment. The expressions of PPARγ protein also exhibited a dose-dependent pattern in response to treatment with either the PPARγ agonist or antagonist (Fig. S7B). The nuclear and cytosol fraction further demonstrated that PPARγ expressions were majorly located in the nucleus and increased when treating with RSG (Fig. 5E, and Supplementary Material 2, Fig. S8). The PPARγ promoter assay also confirmed the additional increase of transcriptional activity when applying RSG during ASC sphere formation (Fig. 5F). The PPARγ agonist further increased the mitochondria complex protein expressions during ASC sphere formation, especially in complex I and III (Fig. S7C). The impact of PPARγ on ASC sphere mitochondrial function was investigated by Seahorse measurements and demonstrated that 10 µM of RSG significantly enhanced OXPHOS in ASC spheres, while 10 µM of GW9662 inhibited sphere-induced OCR and mitochondrial energy production (Fig. 5G). The ROS levels were assessed with the application of PPARγ agonist or antagonist during ASC sphere formation and showed low intracellular ROS when treating with PPARγ agonist (Sph + RSG), whereas the ROS was slightly elevated in ASC sphere with PPARγ antagonist treatment (Sph + GW9662) (Fig. S7D). Taken together, these results confirmed the regulatory mechanism of the EZH2-H3K27me3-PPARγ pathway in ASC sphere formation to enhance mitochondrial function and energy production.

### Sphere-enhanced mitochondria rescue inflamed cells and integrate with endogenous mitochondria

**Fig. 6:**
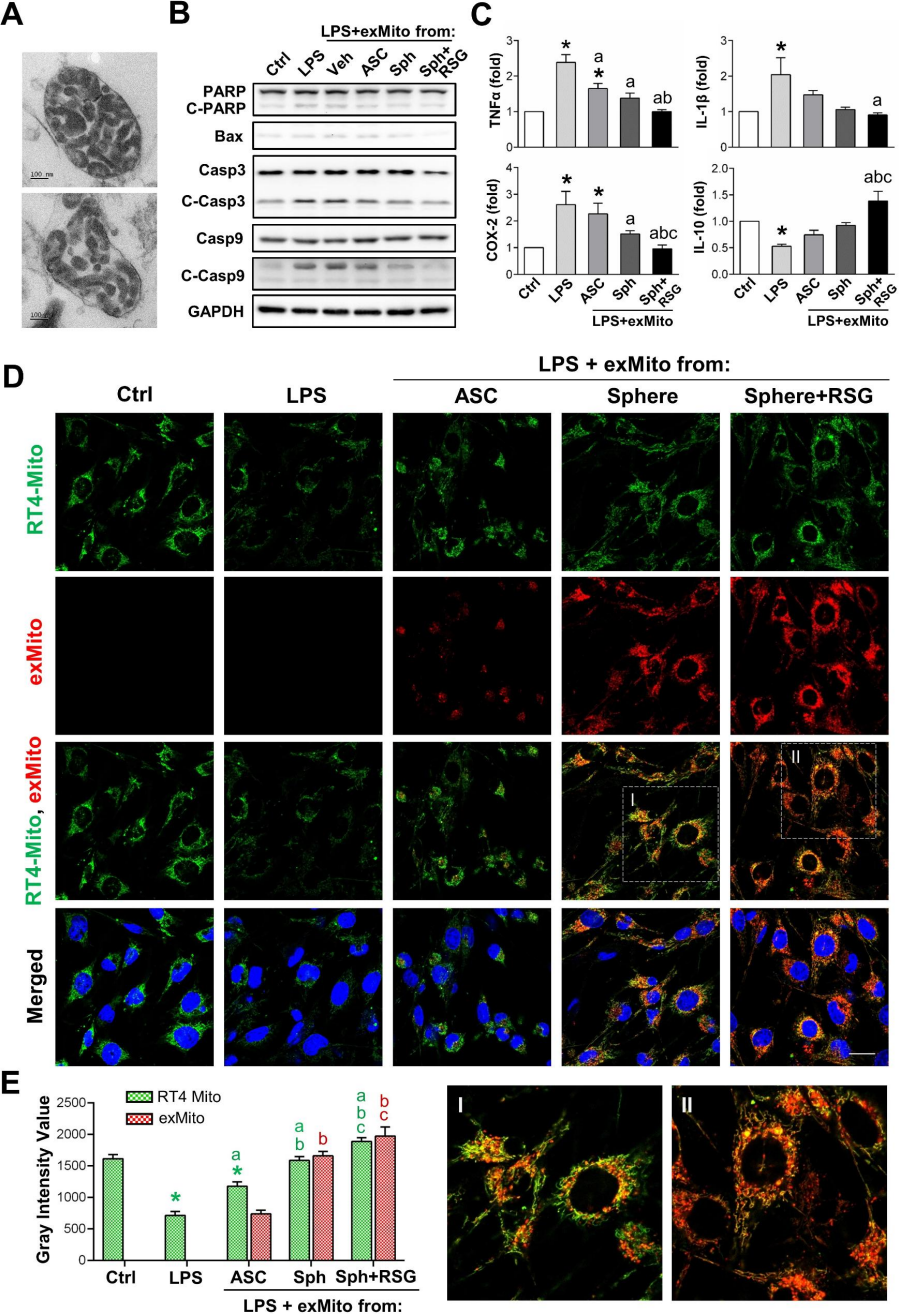
Therapeutic potential of cell-free mitochondria transfer is evidenced by the rescue of inflamed cells through the delivery of sphere-enhanced mitochondria. (**A**) Representative TEM images of mitochondria extracted from ASC spheres after 3 days of seeding ASCs on a chitosan-coated surface. (Magnification: 60000x. Scale bar: 100 nm). (**B**) Representative Western blotting of PARP, cleaved PARP (C-PARP), Caspase-3 (Casp3), cleaved Caspase-3 (C-Casp3), Caspase-9 (Casp9), and cleaved Caspase-9 (C-Casp9) showed the increases of LPS-induced inflammatory and apoptotic protein expression in RT4 cells treated with 20 µg/ml LPS (LPS group). These protein levels were reduced upon treatment with extracted mitochondria (exMito) isolated from 2D-cultured ASCs (ASC), ASC spheres (Sph), or 20 µM RSG-treated ASC spheres (Sph + RSG), which were added to the RT4 cells 3 h after LPS-induced inflammation. The quantification and statistical analyses (*n* = 3) are presented in Supplementary Material 2, Fig. S9A. Full-length blots are presented in Supplementary Material 12, Fig. S14. (**C**) The qPCR analysis of TNFα, IL-1β, IL-6, and IL-10 mRNA expression in RT4 cell Control group, LPS group, and LPS-inflamed groups treated with various exMito (*n* = 4). (**D**) The immunofluorescence images demonstrated the mitochondrial morphologies and expressions of both endogenous mitochondria (RT4-Mito, labeled with MitoTracker Green FM dye) and exMito (labeled in red with MitoTracker Deep Red FM dye) isolated from different ASC groups in the LPS-inflamed RT4 cells. Images I and II represent an enlarged view and their relative positions in the original image. (Scale bar: 20 μm). (**E**) Quantification of fluorescent intensities for endogenous mitochondria (green) and exMito (red) from different ASC inductions demonstrated significant increases in exMito delivery when administering exMito from ASC spheres (Sph group), with a further increase observed when exMito was sourced from the Sph + RSG group (*n* = 3). All values are represented as the mean ± SEM and analyzed by one-way ANOVA with Tukey’s multiple comparisons post-test. *p < 0.05 compared to the RT4 control group (Ctrl). ^a^*p*<0.05 compared to the LPS-induced group (LPS). ^b^*p*<0.05 compared to the LPS + exMito from ASC group. ^c^*p*<0.05 compared to the LPS + exMito from Sph group

In cell-based therapy, our previous research demonstrated that ASC spheres could promote the repair and regeneration of transected sciatic nerves in rats, particularly by inhibiting inflammation and promoting the remyelination in Schwann cells [37, 42]. Given the significant changes in mitochondrial morphology and function during sphere formation, we investigated the therapeutic potential of mitochondria derived from 2D-cultured ASCs and chitosan-induced 3D ASC spheres. To evaluate the anti-inflammatory and cell rescue effects, the mitochondria were freshly isolated from ASCs or ASC spheres and immediately administered to inflamed cells. The TEM images demonstrated that sphere-enhanced mitochondria were easily obtained through the extraction procedures and maintained intact ultrastructure with compacted cristae (Fig. 6A). These sphere-enhanced mitochondria also formed a lipid cluster after isolation.

Cellular inflammation and damage were induced by in vitro applying 20 µg/ml LPS or 10 ng/ml TNFα to rat schwannoma RT4 cells. After 3 h of LPS or TNFα stimulation, freshly extracted mitochondria (exMito) from 2D-cultured ASCs (ASC), chitosan-induced 3D ASC spheres (Sph), and 10 µM RSG-treated ASC spheres (Sph + RSG) were administered to the inflamed cells for 48 h. Apoptosis markers, including cleaved PARP, BAX, cleaved Caspase-3, and cleaved Caspase-9, increased in inflamed RT4 cells under LPS (Fig. 6B, and Supplementary Material 2, Fig. S9A) or TNFα (Fig. S9B) induction. The administration of mitochondria extracted from ASC spheres (Sph) or those treated with a PPARγ agonist (Sph + RSG) rescued cells and reduced the expression of apoptosis-related protein (Fig. 6B and Fig. S9A-B). Pro-inflammatory genes induced by LPS (Fig. 6C) or TNFα (Fig. S9C) at the mRNA level, including TNFα, IL-1β, IL-6, and COX-2, were inhibited following the application of the exMito from ASC spheres with or without RSG (significantly different from LPS-induced RT4 group, marked as ‘a’). Additionally, the administration of exMito from ASC sphere treated with a PPARγ agonist (Sph + RSG) further increased the anti-inflammatory cytokine IL-10 compared to exMito treatment from 2D-cultured ASC (significant difference labeled as ‘b’) and 3D ASC spheres (significant difference labeled as ‘c’) (Fig. 6C). Furthermore, the number and morphology of RT4 cells, which were altered by LPS or TNFα stimulation, were rescued by exMito treatments (Fig. S9D).

To trace and verify the uptake of exMito by inflamed cells, MitoTracker Deep Red FM dye was used to label ASC mitochondria prior to spheroid formation and mitochondrial isolation (exMito labeled in red). MitoTracker Green FM dye was used to label the endogenous mitochondria in RT4 cells to observe the mitochondrial dynamics in response to inflammation and different exMito administrations. After 48 h of LPS-induced inflammation, endogenous mitochondria became fragmented and damaged, as indicated by their shortened morphology (Fig. 6D, RT4-Mito labeled in green). The weakened RT4-Mito fluorescence intensity in LPS-injured cells was rescued by administering any type of exMito from 2D-cultured ASC (ASC) or ASC spheres with or without RSG (Sphere or Sphere + RSG) (Fig. 6E, quantification of green fluorescent intensity compared to LPS group). Endogenous mitochondria in RT4 cells exhibited elongated filamentous morphologies and higher fluorescence when receiving exMito from ASC spheres or spheres with additional RSG (Fig. 6D-E, green fluorescent in the Sph and Sph + RSG groups). Both exMito isolated from the Sph and Sphere + RSG groups showed increased exMito intensities compared to exMito from ASC, indicating higher delivery of sphere-enhanced mitochondria after 3D spheroid formation (Fig. 6D-E, red fluorescent). We also observed that both administered exMito from ASC spheres (both Sph and Sph + RSG groups) merged or were in close proximity to endogenous mitochondria (RT4-Mito), suggesting the possibility of integration or fusion between endogenous and extracted mitochondria (see enlarged images of areas I and II). Quantification of fluorescent intensities in RT4-Mito and exMito demonstrated that the PPARγ agonist (RSG) further improved mitochondrial delivery and transfer into damaged cells to inhibit inflammation (Fig. 6E, with significantly differences labeled ‘b’ or ‘c‘ to indicate comparisons with LPS + exMito from ASC or Sph groups, respectively). The inflammation and rescue by exMito delivery were double-confirmed in a TNFα-induced model (Supplementary Material 2, Fig. S10A-B). Labeling and tracing exMito provide direct evidences to demonstrate how ASCs compact the functional enhanced mitochondria during sphere formation and show better delivery and integration of these enhanced mitochondria to rescue inflammation and repair damaged cells.

**Fig. 7:**
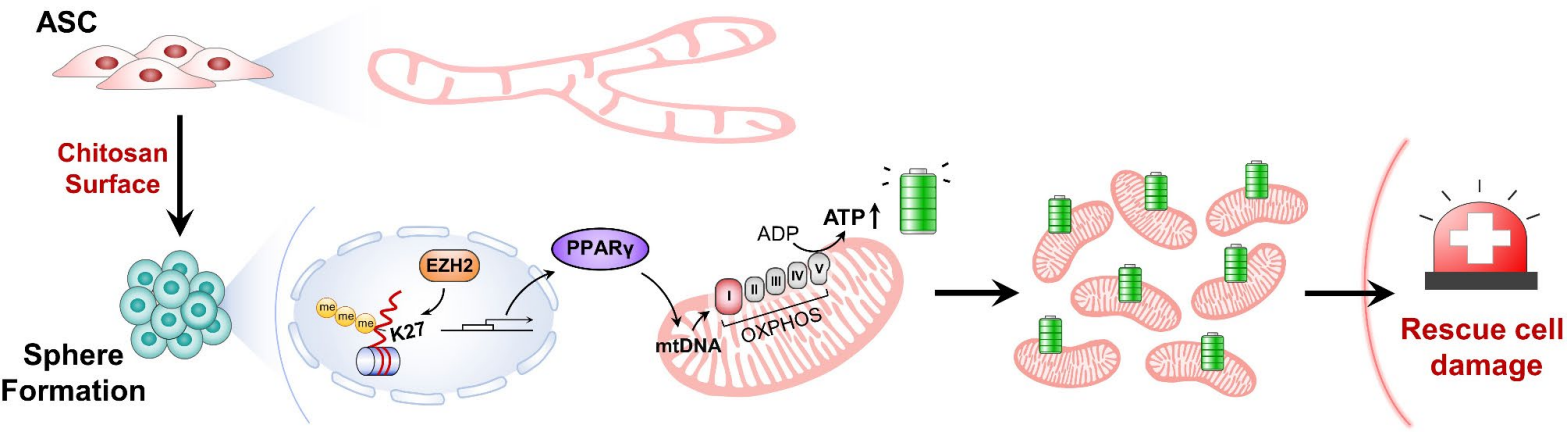
Schematic summary illustrating the enhancement of mitochondrial features, functions, and delivery through the formation 3D ASC spheres using chitosan-coated surfaces. Chitosan-coated surfaces induce ASCs assembly into 3D spheres with enhanced mitochondrial ATP production. Mechanismally, the EZH2-H3K4me3-PPARγ pathway regulates mtDNA associated with mitochondrial complex I in the OXPHOS system. The enhanced mitochondria from ASC spheres exhibit structural stability and higher delivery efficiency, providing an energy source and rescuing damaged endogenous mitochondria, which suggest a potential strategy for mitochondria therapy

Taken together, these results reveal that chitosan-coated surfaces can promote ASCs to form 3D spheres, facilitating the compacted morphological features and enhanced ATP production. These sphere-enhanced mitochondria are regulated via the EZH2-H3K9me3-PPARγ pathway to boost mitochondrial functions. Additionally, sphere-enhanced mitochondria exhibit better structural stability and deliver efficiency to direct transfer into damaged cells for repair and rescue. A schematic outline of the novel discoveries in current study is summarized in Fig. 7.

## Discussion

Mitochondria function is integral to cellular homeostasis and plays a crucial role in various physiological processes, including tissue repair and regeneration. Several studies have highlighted the therapeutic potential of mitochondria transfer in diverse injury and disease models such as acute lung injury [18], cardiac ischemic injury [19], acute myocardial infraction [20], spinal cord injury [21], and stroke [22]. Mitochondria can be incorporated into cells through macropinocytosis and caveolae-dependent endocytosis, eliminating the need for transfection reagents or interventions [25]. Nasal administration of human MSC-derived mitochondria has shown promise in reversing chemotherapy-induced cognitive deficits [32]. Mitochondria isolated from human MSCs and administered nasally can rapidly reached the meninges, internalized by macrophages, and distributed to multiple brain regions for successfully restoring both brain structure and function in cisplatin-induced cognitive deficits mice. Local injection of mitochondria can also restore the fission-impaired or aged satellite cells by improving mitochondrial dynamics (activate fission or prevent fusion), OXPHOS, or mitophagy to promote the regenerative muscle functions [33]. Humans generally exhibit good tolerance to cell-free mitochondria, as evidenced by the presence of cell-free mitochondria in normal blood components [43] and high levels of extracellular mitochondria and mitochondria-associated extracellular vesicles in blood [44]. Although the field of mitochondrial transfer experiments is rapidly advancing, further research and exploration are still needed to understand the detailed mechanisms and potential therapeutic applications of mitochondrial therapy.

Mitochondria not only reflect cellular status in diseases but also play important roles in the functionality of stem or progenitor cells during regeneration. ASCs harvested from type II diabetes patients showed enlarged nuclei, degenerated mitochondrial cristae, and decreased mitochondrial enzyme activity compared to ASCs from healthy donors [45]. The aging or genetic impairment of resident quiescent muscle stem cell (satellite cells) leads to loss of mitochondrial fission, dysregulation of the mitochondrial electron transport chain, inefficient OXPHOS metabolism, increased oxidative stress, and subsequent failure of muscle regeneration [33]. Using scRNA-seq, the mitochondrial membrane potential for glycolysis and metabolic properties were compared between quiescent and cycling-primed hematopoietic stem cell which demonstrated an increased lysosomal sequestration of mitochondria to enhance the competitive repopulation ability and therapeutically relevant in primed cells [46]. Mitochondria are also central regulators of cell fate determination in neural stem cells for neurodevelopment, adult neurogenesis, and cognitive function [47]. The morphology and distribution of mitochondria serve as indicators of mitochondrial cristae development for respiratory chain complex assembly during MSC osteogenic differentiation [48]. During osteogenic differentiation, the changes in MSC mitochondria processes can be inhibited by ROS [49, 50]. Conversely, ROS levels rise as MSCs transform into adipocytes during adipogenesis [51, 52]. In addition, adult stem cells gradually lose their adaptive capacity due to aging and impaired function of energy-producing mitochondria. Enhancing mitochondrial function in stem cells is, therefore, an important area of research. How to boost the mitochondria in stem cell is also an important topic. The current study discovered a convenient strategy to facilitate both mitochondria energy production (Fig. 3) and morphology (Fig. 1) by assembling stem cells into 3D spheres on chitosan-coated surfaces. These features can benefit the high-yield extraction, stable structure during delivery, and the rescue of damaged endogenous mitochondria to repair or restore cell functions (Fig. 6).

In stem cell, the H3K27me3 is critical for maintaining stemness, while H3K27me2 triggers cellular differentiation [53]. Methylation of H3K27 and H3K9 is usually linked to gene silencing, whereas methylation of H3K4 is associated with actively transcribed genes [36]. Our previous study revealed the subpopulations and gene expression profiles in ASC spheres to discover their cell cycle and reprogramming signaling network regulated by epigenetic factors [35]. The interplay between the H3K4me3, H3K9me3, HDAC5 epigenetic modifiers, and their target regulators (KMT2A, SUV39H1, and HDAC5) during chitosan-induced sphere formation was associated with arresting ASC proliferation and promoting of reprogramming potential. The current study further illustrates the pivot role of EZH2 in mitochondrial functions during ASC sphere formation and mitochondria ATP production (Fig. 4). Mechanistically, EZH2, a member of the polycomb repressive complex 2 (PRC2), has multiple splicing modes and performs various physiological functions. Female mice with a short form of EZH2 (Ezh2Short) displayed abnormal mitochondrial function and accumulated H3K27me3 in Ezh2Short oocytes, significantly reducing fertility and obstructing oogenesis [54]. Modulations of EZH2 phosphorylation have shown divergent effects on mitochondrial function, influencing cellular growth, differentiation, and ATP levels [55].

The second major player in sphere-enhanced mitochondria regulatory mechanism is PPARγ, a member of the nuclear receptor superfamily of ligand-inducible transcription factors. PPARγ binds to PPAR-responsive regulatory elements for regulating adipogenesis, lipid metabolism, inflammation, and maintaining metabolic homeostasis [56–58]. PPARGC1A and PPARGC1B are two coactivators of PPARγ that contribute to energy homeostasis and metabolism. PPARγ plays anti-inflammatory roles in obesity, metabolic syndrome, insulin resistance/insufficiency, type 2 diabetes mellitus [58], and inflammation [59–61]. It also been reported to regulate mitochondrial structure and function [62]. The RSG has been reported to reduce diabetes angiopathy by inhibiting mitochondrial dysfunction via regulation of heat shock protein 22 expression [63]. The addition of a PPARγ agonist or antagonist during ASC sphere formation revealed that the PPARγ signal can altered sphere size (Fig. 5D) and further improve ATP production in ASC spheres by adding RSG during spheroid formation (Fig. 5G). The interactions between the epigenetic regulator and the PPARγ signal were predicted by IPA in current study (Fig. 4A) and confirmed by molecular measurements to demonstrate that PPARγ and EZH2 can influence each other (Fig. 5A, C, and E). Blockage of the relative signaling network with the EZH2 inhibitor GSK126 confirmed the epigenetic regulation of H3K27me3 and its downstream targets of PPARγ and mitochondrial genes (Fig. 5A and C). Although RSG treatment increased PPARγ signaling during sphere formation and further boosted the therapeutic potential of mitochondria, RSG is commonly found in adipogenesis medium for MSC differentiation into adipocytes. The long-term effects of RSG treatment during ASC spheroid formation and its influence on differentiation markers require further investigation in future studies. Thus, the current study revealed that the EZH2-H3K27me3-PPARγ regulatory pathway plays an important role in sphere-enhanced mitochondria functions during ASC spheroid formation.

Changes in the microenvironment directly influence cellular metabolic processes, subsequently affecting mitochondrial function. Sphere formations alter mitochondrial morphology, especially in cells deprived of oxygen and nutrients at the sphere center [64]. Moreover, a detailed understanding of cell-cell and cell-microenvironment interactions in spheres, particularly regarding epigenetic regulation for mitochondria function, has not been investigated previously. This study utilized scRNA-seq to provide insights into the epigenetic regulatory mechanisms during sphere formation and discovered a special subpopulation (Cluster 4) that promotes the mitochondrial function and ATP production (Figs. 2 and 3). Although the underlying mechanism and potential mechanobiological factors that control the change of mitochondrial morphology during 3D stem cell assembly remain unknown, our unpublished data observed some association with cytoskeleton rearrangements during sphere formation. For the extracted mitochondria and potential mitochondrial therapy, future studies can focus on tracing these donor mitochondria (exMito) and elucidating the potential mechanism in the in vivo repair and regenerative microenvironments in living animals.

## Conclusion

Through scRNA-seq and rigorous cell fate trajectory analyses, we identified distinct cell subpopulations enriched in mitochondrial functions within spheres formed by seeding ASCs on chitosan-coated surface. Cluster 4 cells are regulated via the EZH2-H3K27me3-PPARγ pathway to upregulate mitochondria complex I genes for producing ATP in spheres. Administration of extracted mitochondria from ASC spheres successfully rescued the LPS-induced cell damage and inflammation. External high-ATP mitochondria were taken up by injured cells and integrated with endogenous mitochondria to restore mitochondrial morphology and functions. The supplementation of the PPARγ agonist Rosiglitazone during sphere formation further increased the ATP production and boosted the rescue capacity of mitochondria therapy. These innovative discoveries and strategies may provide potential therapeutic applications in regenerative medicine and clinical interventions.

## Electronic supplementary material

Below is the link to the electronic supplementary material.

Supplementary Material 1

Supplementary Material 2

Supplementary Material 3

Supplementary Material 4

Supplementary Material 5

Supplementary Material 6

Supplementary Material 7

Supplementary Material 8

Supplementary Material 9

Supplementary Material 10

Supplementary Material 11

Supplementary Material 12

Supplementary Material 13

## Data Availability

All data are available in the main text or the supplementary materials. Additional experimental details and data used or analyzed during the current study can be obtained from the corresponding author upon reasonable request. The scRNA-seq data reported in this article has been deposited in NCBI’s Gene Expression Omnibus (GEO) under accession number GSE250358. The data is currently private and is scheduled to be released on December 16, 2027, or upon the publication of this manuscript.

## Abbreviations

ASC: Adipose-derived stem cells
CS: Chitosan-coated surface
ECAR: Extracellular acidification rates
exMito: Extracted mitochondria
EZH2: Enhancer of zeste 2 polycomb repressive complex 2 subunit
FBS: Fetal bovine serum
FN: Fibronectin
H3K4me3: Histone 3 lysine 4 trimethylation
H3K9me3: Histone 3 lysine 9 trimethylation
H3K27me3: Histone 3 lysine 27 trimethylation
HDAC5: Histone deacetylase 5
IPA: Ingenuity Pathway Analysis software
LPS: Lipopolysaccharide
MSC: Mesenchymal stem cells
mtDNA: Mitochondrial DNA
FCCP: Carbonylcyanide-p-trifluoromethoxyphenylhydrazone
KMT2A: Histone-lysine N-methyltransferase 2 A
OCR: Oxygen consumption rate
OXPHOS: Oxidative phosphorylation
PKM: Pyruvate kinase M1/2
PPARγ/PPRG: Peroxisome proliferator-activated receptor gamma
PPARGC1A: PPARG coactivator 1 alpha
PPARGC1B: PPARG coactivator 1 beta
ROS: Reactive oxygen species
RSG: Rosiglitazone
scRNA-seq: Single-cell RNA sequencing)
Sph: Spheres
SREBF1: Sterol regulatory element-binding protein 1
STAT3: Signal transducer and activator of transcription 3
TEF3: Transcription factor for Immunoglobulin Heavy-Chain Enhancer 3
TEM: Transmission Electron Microscopy
TFAM: Mitochondrial transcription factor A
TNFα: Tumor Necrosis Factor-α
ULA: Ultra-low attachment plate

## References

[1] Aly RM. Current state of stem cell-based therapies: an overview. Stem Cells Int. 2020;7:8. 10.21037/sci-2020-001 PMC7367472 32695801

[2] Pittenger MF, Discher DE, Peault BM, Phinney DG, Hare JM, Caplan AI. Mesenchymal stem cell perspective: cell biology to clinical progress. NPJ Regen Med. 2019;4:22. 31815001 10.1038/s41536-019-0083-6 PMC6889290

[3] Zuk PA. The adipose-derived stem cell: looking back and looking ahead. Mol Biol Cell. 2010;21(11):1783–7. 20375149 10.1091/mbc.E09-07-0589 PMC2877637

[4] Goldrick C, Guri I, Herrera-Oropeza G, O’Brien-Gore C, Roy E, Wojtynska M, et al. 3D multicellular systems in disease modelling: from organoids to organ-on-chip. Front Cell Dev Biol. 2023;11:1083175. 36819106 10.3389/fcell.2023.1083175 PMC9933985

[5] Vogt N, Assembloids. Nat Methods. 2021;18(1):27. 10.1038/s41592-020-01026-x 33408387

[6] Ryu NE, Lee SH, Park H. Spheroid Culture System methods and applications for mesenchymal stem cells. Cells. 2019;8(12). 10.3390/cells8121620 PMC6953111 31842346

[7] Cheng NC, Chen SY, Li JR, Young TH. Short-term spheroid formation enhances the regenerative capacity of adipose-derived stem cells by promoting stemness, angiogenesis, and chemotaxis. Stem Cells Transl Med. 2013;2(8):584–94. 23847001 10.5966/sctm.2013-0007 PMC3726138

[8] Ho SS, Murphy KC, Binder BY, Vissers CB, Leach JK. Increased survival and function of mesenchymal stem cell spheroids entrapped in instructive Alginate Hydrogels. Stem Cells Transl Med. 2016;5(6):773–81. 27057004 10.5966/sctm.2015-0211 PMC4878334

[9] Yeh HY, Hsieh FY, Hsu SH. Self-patterning of adipose-derived mesenchymal stem cells and chondrocytes cocultured on hyaluronan-grafted chitosan surface. Biointerphases. 2016;11(1):011011. 26916660 10.1116/1.4942754

[10] Huang CF, Chang YJ, Hsueh YY, Huang CW, Wang DH, Huang TC, et al. Assembling composite dermal papilla spheres with adipose-derived stem cells to enhance hair follicle induction. Sci Rep. 2016;6:26436. 27210831 10.1038/srep26436 PMC4876394

[11] Borcherding N, Jia W, Giwa R, Field RL, Moley JR, Kopecky BJ, et al. Dietary lipids inhibit mitochondria transfer to macrophages to divert adipocyte-derived mitochondria into the blood. Cell Metab. 2022;34(10):1499–513. e8. 36070756 10.1016/j.cmet.2022.08.010 PMC9547954

[12] Tilokani L, Nagashima S, Paupe V, Prudent J. Mitochondrial dynamics: overview of molecular mechanisms. Essays Biochem. 2018;62(3):341–60. 30030364 10.1042/EBC20170104 PMC6056715

[13] Fenton AR, Jongens TA, Holzbaur ELF. Mitochondrial dynamics: shaping and remodeling an organelle network. Curr Opin Cell Biol. 2021;68:28–36. 32961383 10.1016/j.ceb.2020.08.014 PMC7925334

[14] Norat P, Soldozy S, Sokolowski JD, Gorick CM, Kumar JS, Chae Y, et al. Mitochondrial dysfunction in neurological disorders: exploring mitochondrial transplantation. Npj Regen Med. 2020;5(1):22. 33298971 10.1038/s41536-020-00107-x PMC7683736

[15] Zhang S, Zhao J, Quan Z, Li H, Qing H. Mitochondria and other organelles in neural development and their potential as therapeutic targets in neurodegenerative diseases. Front Neurosci. 2022;16:853911. 35450015 10.3389/fnins.2022.853911 PMC9016280

[16] Westermann B. Mitochondrial fusion and fission in cell life and death. Nat Rev Mol Cell Biol. 2010;11(12):872–84. 21102612 10.1038/nrm3013

[17] Youle RJ, van der Bliek AM. Mitochondrial fission, fusion, and stress. Science. 2012;337(6098):1062–5. 22936770 10.1126/science.1219855 PMC4762028

[18] Islam MN, Das SR, Emin MT, Wei M, Sun L, Westphalen K, et al. Mitochondrial transfer from bone-marrow-derived stromal cells to pulmonary alveoli protects against acute lung injury. Nat Med. 2012;18(5):759–65. 22504485 10.1038/nm.2736 PMC3727429

[19] Han H, Hu J, Yan Q, Zhu J, Zhu Z, Chen Y, et al. Bone marrow-derived mesenchymal stem cells rescue injured H9c2 cells via transferring intact mitochondria through tunneling nanotubes in an in vitro simulated ischemia/reperfusion model. Mol Med Rep. 2016;13(2):1517–24. 26718099 10.3892/mmr.2015.4726 PMC4732861

[20] Kaza AK, Wamala I, Friehs I, Kuebler JD, Rathod RH, Berra I, et al. Myocardial rescue with autologous mitochondrial transplantation in a porcine model of ischemia/reperfusion. J Thorac Cardiovasc Surg. 2017;153(4):934–43. 27938904 10.1016/j.jtcvs.2016.10.077

[21] Lin MW, Fang SY, Hsu JC, Huang CY, Lee PH, Huang CC, et al. Mitochondrial transplantation attenuates neural damage and improves locomotor function after traumatic spinal cord Injury in rats. Front Neurosci. 2022;16:800883. 35495036 10.3389/fnins.2022.800883 PMC9039257

[22] Zhang Z, Ma Z, Yan C, Pu K, Wu M, Bai J, et al. Muscle-derived autologous mitochondrial transplantation: a novel strategy for treating cerebral ischemic injury. Behav Brain Res. 2019;356:322–31. 30213662 10.1016/j.bbr.2018.09.005

[23] Jiang G, Jiang T, Chen J, Yao H, Mao R, Yang X, et al. Mitochondrial dysfunction and oxidative stress in diabetic wound. J Biochem Mol Toxicol. 2023;37(7):e23407. 37341017 10.1002/jbt.23407

[24] Borcherding N, Brestoff JR. The power and potential of mitochondria transfer. Nature. 2023;623(7986):283–91. 37938702 10.1038/s41586-023-06537-z PMC11590279

[25] Main EN, Cruz TM, Bowlin GL. Mitochondria as a therapeutic: a potential new frontier in driving the shift from tissue repair to regeneration. Regen Biomater. 2023;10:rbad070. 37663015 10.1093/rb/rbad070 PMC10468651

[26] Lin W, Chen S, Wang Y, Wang M, Lee WY-W, Jiang X, et al. Dynamic regulation of mitochondrial-endoplasmic reticulum crosstalk during stem cell homeostasis and aging. Cell Death Dis. 2021;12(9):794. 34400615 10.1038/s41419-021-03912-4 PMC8368094

[27] Atashi F, Modarressi A, Pepper MS. The role of reactive oxygen species in mesenchymal stem cell adipogenic and osteogenic differentiation: a review. Stem Cells Dev. 2015;24(10):1150–63. 25603196 10.1089/scd.2014.0484 PMC4424969

[28] Naik PP, Birbrair A, Bhutia SK. Mitophagy-driven metabolic switch reprograms stem cell fate. Cell Mol Life Sci. 2019;76(1):27–43. 30267101 10.1007/s00018-018-2922-9 PMC11105479

[29] Yan W, Diao S, Fan Z. The role and mechanism of mitochondrial functions and energy metabolism in the function regulation of the mesenchymal stem cells. Stem Cell Res Ther. 2021;12(1):140. 33597020 10.1186/s13287-021-02194-z PMC7890860

[30] Quinn KP, Sridharan GV, Hayden RS, Kaplan DL, Lee K, Georgakoudi I. Quantitative metabolic imaging using endogenous fluorescence to detect stem cell differentiation. Sci Rep. 2013;3:3432. 24305550 10.1038/srep03432 PMC3851884

[31] Malekpour K, Hazrati A, Soudi S, Hashemi SM. Mechanisms behind therapeutic potentials of mesenchymal stem cell mitochondria transfer/delivery. J Control Release. 2023;354:755–69. 36706838 10.1016/j.jconrel.2023.01.059

[32] Alexander JF, Seua AV, Arroyo LD, Ray PR, Wangzhou A, Heibeta-Luckemann L, et al. Nasal administration of mitochondria reverses chemotherapy-induced cognitive deficits. Theranostics. 2021;11(7):3109–30. 33537077 10.7150/thno.53474 PMC7847685

[33] Hong X, Isern J, Campanario S, Perdiguero E, Ramirez-Pardo I, Segales J, et al. Mitochondrial dynamics maintain muscle stem cell regenerative competence throughout adult life by regulating metabolism and mitophagy. Cell Stem Cell. 2022;29(9):1298–314. e10. 35998641 10.1016/j.stem.2022.07.009

[34] Cheng XT, Huang N, Sheng ZH. Programming axonal mitochondrial maintenance and bioenergetics in neurodegeneration and regeneration. Neuron. 2022;110(12):1899–923. 35429433 10.1016/j.neuron.2022.03.015 PMC9233091

[35] Chang MM, Hong YK, Hsu CK, Harn HI, Huang BM, Liu YH, et al. Histone trimethylations and HDAC5 regulate spheroid subpopulation and differentiation signaling of human adipose-derived stem cells. Stem Cells Transl Med. 2024;13(3):293–308. 38173411 10.1093/stcltm/szad090 PMC10940829

[36] Volker-Albert M, Bronkhorst A, Holdenrieder S, Imhof A. Histone modifications in Stem Cell Development and their clinical implications. Stem Cell Rep. 2020;15(6):1196–205. 10.1016/j.stemcr.2020.11.002 PMC7724464 33296672

[37] Huang CW, Lu SY, Huang TC, Huang BM, Sun HS, Yang SH, et al. FGF9 induces functional differentiation to Schwann cells from human adipose derived stem cells. Theranostics. 2020;10(6):2817–31. 32194837 10.7150/thno.38553 PMC7052907

[38] Qiu X, Mao Q, Tang Y, Wang L, Chawla R, Pliner HA, et al. Reversed graph embedding resolves complex single-cell trajectories. Nat Methods. 2017;14(10):979–82. 28825705 10.1038/nmeth.4402 PMC5764547

[39] Huang TC, Wu HL, Chen SH, Wang YT, Wu CC. Thrombomodulin facilitates peripheral nerve regeneration through regulating M1/M2 switching. J Neuroinflamm. 2020;17(1):240. 10.1186/s12974-020-01897-z PMC7477856 32799887

[40] Casanova A, Wevers A, Navarro-Ledesma S, Pruimboom L. Mitochondria: it is all about energy. Front Physiol. 2023;14:1114231. 37179826 10.3389/fphys.2023.1114231 PMC10167337

[41] Li Q, Gao Z, Chen Y, Guan MX. The role of mitochondria in osteogenic, adipogenic and chondrogenic differentiation of mesenchymal stem cells. Protein Cell. 2017;8(6):439–45. 28271444 10.1007/s13238-017-0385-7 PMC5445026

[42] Hsueh YY, Chang YJ, Huang TC, Fan SC, Wang DH, Chen JJ, et al. Functional recoveries of sciatic nerve regeneration by combining chitosan-coated conduit and neurosphere cells induced from adipose-derived stem cells. Biomaterials. 2014;35(7):2234–44. 24360575 10.1016/j.biomaterials.2013.11.081

[43] Trumpff C, Michelson J, Lagranha CJ, Taleon V, Karan KR, Sturm G, et al. Stress and circulating cell-free mitochondrial DNA: a systematic review of human studies, physiological considerations, and technical recommendations. Mitochondrion. 2021;59:225–45. 33839318 10.1016/j.mito.2021.04.002 PMC8418815

[44] Byappanahalli AM, Omoniyi V, Noren Hooten N, Smith JT, Mode NA, Ezike N, et al. Extracellular vesicle mitochondrial DNA levels are associated with race and mitochondrial DNA haplogroup. iScience. 2024;27(1):108724. 38226163 10.1016/j.isci.2023.108724 PMC10788249

[45] Horiguchi M, Hata S, Tsurudome Y, Ushijima K. Characterizing the degeneration of nuclear membrane and mitochondria of adipose-derived mesenchymal stem cells from patients with type II diabetes. J Cell Mol Med. 2021;25(9):4298–306. 33759360 10.1111/jcmm.16484 PMC8093988

[46] Liang R, Arif T, Kalmykova S, Kasianov A, Lin M, Menon V, et al. Restraining lysosomal activity preserves hematopoietic stem cell quiescence and potency. Cell Stem Cell. 2020;26(3):359–76. e7. 32109377 10.1016/j.stem.2020.01.013 PMC8075247

[47] Khacho M, Harris R, Slack RS. Mitochondria as central regulators of neural stem cell fate and cognitive function. Nat Rev Neurosci. 2019;20(1):34–48. 30464208 10.1038/s41583-018-0091-3

[48] Kasahara A, Scorrano L. Mitochondria: from cell death executioners to regulators of cell differentiation. Trends Cell Biol. 2014;24(12):761–70. 25189346 10.1016/j.tcb.2014.08.005

[49] Chen CT, Shih YR, Kuo TK, Lee OK, Wei YH. Coordinated changes of mitochondrial biogenesis and antioxidant enzymes during osteogenic differentiation of human mesenchymal stem cells. Stem Cells. 2008;26(4):960–8. 18218821 10.1634/stemcells.2007-0509

[50] Yoon H, Park SG, Kim HJ, Shin HR, Kim KT, Cho YD, et al. Nicotinamide enhances osteoblast differentiation through activation of the mitochondrial antioxidant defense system. Exp Mol Med. 2023;55(7):1531–43. 37464093 10.1038/s12276-023-01041-w PMC10393969

[51] Comas F, Latorre J, Ortega F, Oliveras-Canellas N, Lluch A, Ricart W, et al. Permanent cystathionine-beta-synthase gene knockdown promotes inflammation and oxidative stress in immortalized human adipose-derived mesenchymal stem cells, enhancing their adipogenic capacity. Redox Biol. 2021;42:101668. 32800520 10.1016/j.redox.2020.101668 PMC8113015

[52] Higuchi M, Dusting GJ, Peshavariya H, Jiang F, Hsiao ST, Chan EC, et al. Differentiation of human adipose-derived stem cells into fat involves reactive oxygen species and forkhead box O1 mediated upregulation of antioxidant enzymes. Stem Cells Dev. 2013;22(6):878–88. 23025577 10.1089/scd.2012.0306 PMC3585477

[53] Bernstein BE, Mikkelsen TS, Xie X, Kamal M, Huebert DJ, Cuff J, et al. A bivalent chromatin structure marks key developmental genes in embryonic stem cells. Cell. 2006;125(2):315–26. 16630819 10.1016/j.cell.2006.02.041

[54] Guo SM, Liu XP, Tian Q, Fei CF, Zhang YR, Li ZM, et al. Regulatory roles of alternative splicing at Ezh2 gene in mouse oocytes. Reprod Biol Endocrinol. 2022;20(1):99. 35791029 10.1186/s12958-022-00962-x PMC9254527

[55] Ozes AR, Pulliam N, Ertosun MG, Yilmaz O, Tang J, Copuroglu E, et al. Protein kinase A-mediated phosphorylation regulates STAT3 activation and oncogenic EZH2 activity. Oncogene. 2018;37(26):3589–600. 29576612 10.1038/s41388-018-0218-z PMC6023775

[56] Ahmadian M, Suh JM, Hah N, Liddle C, Atkins AR, Downes M, et al. PPARgamma signaling and metabolism: the good, the bad and the future. Nat Med. 2013;19(5):557–66. 23652116 10.1038/nm.3159 PMC3870016

[57] Rosen ED, Sarraf P, Troy AE, Bradwin G, Moore K, Milstone DS, et al. PPAR gamma is required for the differentiation of adipose tissue in vivo and in vitro. Mol Cell. 1999;4(4):611–7. 10549292 10.1016/s1097-2765(00)80211-7

[58] Wu D, Eeda V, Undi RB, Mann S, Stout M, Lim HY, et al. A novel peroxisome proliferator-activated receptor gamma ligand improves insulin sensitivity and promotes browning of white adipose tissue in obese mice. Mol Metab. 2021;54:101363. 34710641 10.1016/j.molmet.2021.101363 PMC8627988

[59] Mierzejewski K, Paukszto L, Kurzynska A, Kunicka Z, Jastrzebski JP, Makowczenko KG, et al. PPARgamma regulates the expression of genes involved in the DNA damage response in an inflamed endometrium. Sci Rep. 2022;12(1):4026. 35256739 10.1038/s41598-022-07986-8 PMC8901773

[60] Cataldi S, Aprile M, Melillo D, Mucel I, Giorgetti-Peraldi S, Cormont M et al. TNFalpha mediates inflammation-Induced effects on PPARG Splicing in Adipose tissue and mesenchymal precursor cells. Cells. 2021;11(1). 10.3390/cells11010042 PMC8750445 35011604

[61] Landi S, Moreno V, Gioia-Patricola L, Guino E, Navarro M, de Oca J, et al. Association of common polymorphisms in inflammatory genes interleukin (IL)6, IL8, tumor necrosis factor alpha, NFKB1, and peroxisome proliferator-activated receptor gamma with colorectal cancer. Cancer Res. 2003;63(13):3560–6. 12839942

[62] Yeligar SM, Kang BY, Bijli KM, Kleinhenz JM, Murphy TC, Torres G, et al. PPARgamma regulates mitochondrial structure and function and human pulmonary artery smooth muscle cell proliferation. Am J Respir Cell Mol Biol. 2018;58(5):648–57. 29182484 10.1165/rcmb.2016-0293OC PMC5946324

[63] Yu L, Chen S, Liang Q, Huang C, Zhang W, Hu L, et al. Rosiglitazone reduces diabetes angiopathy by inhibiting mitochondrial dysfunction dependent on regulating HSP22 expression. iScience. 2023;26(4):106194. 36968091 10.1016/j.isci.2023.106194 PMC10031002

[64] Tan Y, Richards D, Coyle RC, Yao J, Xu R, Gou W, et al. Cell number per spheroid and electrical conductivity of nanowires influence the function of silicon nanowired human cardiac spheroids. Acta Biomater. 2017;51:495–504. 28087483 10.1016/j.actbio.2017.01.029 PMC5346043

