# Supplementary material for: Enhanced mitochondrial function and delivery from adipose-derived stem cell spheres via the EZH2-H3K27me3-PPARγ pathway for advanced therapy
Source: Stem Cell Res Ther. 2025 Mar 11;16:129. doi: 10.1186/s13287-025-04164-1 (PMC11899936; doi:10.1186/s13287-025-04164-1)
Supplement: Supplementary file 1 — Supplementary Material 1 [file 13287_2025_4164_MOESM1_ESM.pdf]

Table S1. Chemicals and materials used in this study.

Chemical / Material	Manufacturer[a]	Catalogue No.
2-ME (2-Mercaptoethanol)	Sigma-Aldrich	M6250
10x Genomics 3' CellPlex Kit	10x Genomics	PN-1000268
ATP determination kit	Thermo Fisher Scientific	A222066
Acrylamide/Bis-acrylamide, 30% solution	Sigma-Aldrich	A3574-100ML
Human mesenchymal stem cells (hMSC) derived from adipose tissue, cryopreserved	PromoCell	C-12977
Bovine serum albumin (BSA)	UniRegion BioTech	UR-BSA001-100G
Chitosan (low molecular weight) Deacetylated chitin, Poly (D-glucosamine)	Sigma-Aldrich	448869-250G
DMEM powder, high glucose Dulbecco's modified eagle medium	Gibco / Thermo Fisher Scientific	12400-061
DMSO	Sigma-Aldrich	D4540-500ML
Ethanol	J.T.Baker	8006-05
Fetal bovine serum (FBS)	HyClone/ GE Healthcare Life Sciences	SH30396.03 (AF29545069)
Glycine	J.T Baker	4059-06
Hs68 cells	BCRC	60038
Hydrochloric acid (HCl)	Merck	K49418617738
Inhibitors		
Chaetocin (SUV39H1 inhibitor)	Cayman Chemical	13156
GSK126 (EZH2 inhibitor)	MCE	HY-13470
LMK235 (HDAC5 inhibitor)	Cayman Chemical	14969
Revumenib (SNDX-5613)	MCE	HY-136175
Live cell image solution	Thermo Fisher Scientific	A14291DJ

Table S1. Chemicals and materials used in this study (continued).

Chemical / Material	Manufacturer[a]	Catalogue No.
Mesenchymal Stem cell Growth Medium 2	PromoCell	C28009
Methanol	DUKSAN	62
Mitochondria Isolation Kit for Culture Cells	Abcam	Ab110171
MitoTracker™ Deep Red FM	Thermo Fisher Scientific	M22426
MitoTracker™ Green FM	Thermo Fisher Scientific	M7514
NucBlue™ Live ReadyProbes™ Reagent (Hoechst 33342)	Thermo Fisher Scientific	R37605
Nuclear extraction kit	Cayman	10009277
Paraformaldehyde 8% Aqueous Solution, EM Grade	Electron Microscopy Sciences, USA	157-8
Penicillin/Streptomycin	Gibco / Thermo Fisher Scientific	CC502-0100
Potassium chloride (KCl)	Sigma-Aldrich	P5405-250G
Potassium dihydrogen phosphate (KH_2PO_4)	Sigma-Aldrich	P5655-100G
PPARγ agonist: Rosiglitazone (RSG)	Cayman Chemical	71740
PPARγ antagonist: GW9662	Cayman Chemical	70785
PPARγ transcription factor assay kit	Abcam	ab133101
ProLong® Diamond Antifade Mountant with DAPI	Thermo Fisher Scientific	P36962
Protease inhibitor cocktail	Sigma-Aldrich	P8340-1ML

Table S1. Chemicals and materials used in this study (continued).

Chemical / Material	Manufacturer[a]	Catalogue No.
Protein quantification assay dye	BioRad Laboratories	5000006
PVDF membrane (Polyvinylidence difluoride membrane; 0.22 micron pore size)	PALL Life Science	BSP0161
Rhodamine phalloidin	Thermo Fisher Scientific	R415
Cellular ROS assay kit	Abcam	Ab113851
Seahorse XF cell mito stress test kit	Agilent Technologies	XFR103015-100
Seahorse XF real-time ATP rate assay kit	Agilent Technologies	XFR103592-100
Seahorse XF DMRM assay medium pack	Agilent Technologies	XFR103680-100
Seahorse XFe24 FluxPak	Agilent Technologies	XFR102340-100
Seahorse XFe24 Islet Capture FluxPak	Agilent Technologies	XFR103518-100
Sodium bicarbonate ($NaHCO_3$)	Sigma-Aldrich	S5761-500G
Disodium hydrogen phosphate (Na_2HPO_4)	Sigma-Aldrich	S5136-100G
Sodium azide (NaN_3)	Sigma-Aldrich	S2002-100G
Sodium chloride (NaCl)	Sigma-Aldrich	S6191-1KG
Sodium dodecyl sulfate (SDS)	SERVA	20765.03
Sodium hydroxide (NaOH)	Sigma-Aldrich	S2770
Sodium orthovanadate (Na_3VO_4)	Sigma-Aldrich	S6508
TBE buffer (5x)	UniRegion Bio Tech	UR-TBEL

Table S1. Chemicals and materials used in this study (continued).

Chemical / Material	Manufacturer[a]	Catalogue No.
Tris base	J.T. Baker	4109-06
Tris HCl	J.T. Baker	4103-02
Triton X-100	GERBU	2000
Trypen blue	Sigma-Aldrich	T8154
Trypsin-EDTA (0.5%, 10x)	Gibco / Thermo Fisher Scientific	15400-054
Tween 20	PanReac AppliChem	123412-1611
Trident femto Western HRP Substrate	GeneTex	GTX14698
Ultra-low attachment microplate, clear round bottom, 96 wells	Corning	7007

[a] The headquarters locations of companies: **10x Genomics**, Pleasanton, CA, USA; **Abcam**, Cambridge, UK; **Agilent Technologies**, Santa Clara, CA, USA; **BCRC** (Bioresource Collection and Research Center), Hsinchu, Taiwan; **BD** (Becton, Dickinson and Company), Franklin Lakes, NJ, USA; **Cayman Chemical**, Ann Arbor, MI, USA; **Corning Inc.,** NY, USA**; DUKSAN**, Ansan City, Kyunggi, Korea; **Electron Microscopy Sciences,** Hatfield, PA, USA **GE Healthcare Life Sciences**, South Logan, UT, USA; **GeneTex**, Hsinchu, Taiwan; **Gibco**, Grand Island, NY, USA; **GERBU** Biotechnik GmbH, Heidelberg, German; **J.T.Baker**/Fisher Scientific UK Ltd., Loughborough, UK; **MCE** (MedChemExpress), Monmouth Junction, NJ , USA; **Merck,** Darmstadt, Germany; **PALL Corporation**, Port Washington, NY, USA; **PromoCell GmbH**, Heidelberg, Germany; **Sigma-Aldrich**, St. Louis, MO, USA; **SERVA** Electrophoresis GmbH, Heidelberg, German; **Thermo Fisher Scientific Inc.**, Waltham, MA, USA; **UniRegion Bio Tech**, Taipei, Taiwan.

Table S2. Antibodies used in this study.

Antibody	Manufacturer[a]	Catalogue No.	Species/Clonality	Application[b]
**Primary Antibody**				
BCL-2	Cell Signaling	2876	rabbit/polyclonal	WB
BAX	Cell Signaling	2772	rabbit/polyclonal	WB
Caspase-3/p17/p19	Proteintech	19677-1-AP	rabbit/polyclonal	WB
Caspase-9	Cell Signaling	9508	mouse/monoclonal	WB
EZH2	Proteintech	21800-1-AP	rabbit/monoclonal	WB
GAPDH	Proteintech	10494-1-AP	rabbit/polyclonal	WB
H3K4me3	Abcam	ab213224	rabbit/monoclonal	WB
H3K9me3	Abcam	ab176916	rabbit/monoclonal	WB
H3K27me3	Abcam	ab192985	rabbit/monoclonal	WB
HDAC5	Cell Signaling	20458	rabbit/monoclonal	WB
Phospho-HDAC4/5/7	Abcam	ab240643	rabbit/monoclonal	WB
KMT2A	Cell Signaling	14197	rabbit/monoclonal	WB
Total OXPHOS cocktail	Abcam	ab110413	mouse/monoclonal	WB
PPARγ	Cell Signaling	2435	rabbit/monoclonal	WB
PARP	Cell Signaling	9542	rabbit/polyclonal	WB
STAT3	BD	810189	mouse/monoclonal	WB
Phospho-STAT3 (Tyr705)	GeneTex	GTX118000	rabbit/polyclonal	WB

Table S2. Antibodies used in this study (continued).

Antibody	Manufacturer[a]	Catalogue No.	Species/Clonality	Application[b]
IgG, whole molecule from normal serum	Jackson ImmunoResearch	005-000-003	goat	Negative control for all experiments
		015-000-003	mouse	
		011-000-003	rabbit	
**Secondary Antibody**				
Anti-mouse IgG HRP-conjugated	Cell Signaling	7076S	Goat	WB
Anti-rabbit IgG HRP-coniugated	Cell Signaling	7074S	`Goat	WB

The headquarters locations of companies: **Abcam**, Cambridge, UK; **Cell Signaling**, Beverly, MA, USA; **GeneTex**, Irvine, CA, USA; **Jackson ImmunoResearch,** West Grove, PA, USA; **Proteintech**, Rosemont, IL, USA; **Sigma-Aldrich**, St. Louis, MO, USA.

[b]WB, western blot analysis.

Table S3. Real-time PCR primers used in this study.

Gene[1]	Primer sequence	Gene[1]	Primer sequence
hCOX2	F: 5’-CCCTTCTGCCTGACACCTTT-3’<br>R: 5’-TTCTGTACTGCGGGTGGAAC-3’	hSTAT3	F: 5’-GAGGACTGAGCATCGAGCAG-3’<br>R: 5’-GTTCCAAAGGGCCAGGATGTA-3’
hGAPDH	F:5’-GTCTCCTCTGACTTCAACAGCG-3<br>R:5’-ACCCTGTTGCTGTAGCCAAAT-3	hTFAM	F: 5’-TTAGAAGAATTGCCCAGCGT-3’<br>R: 5’-CACTCCGCCCTATAAGCATC-3’
hIL-1β	F: 5’-GTACCTGTCCTGCGTGTTGA-3’<br>R: 5’-GGGAACTGGGCAGACTCAAA-3’	hTNFα	F: 5’-AAGCCTGTAGCCCATGTTGT-3’<br>R: 5’-GAGGTACAGGCCCTCTGATG-3’
hIL-6	F: 5’-AGTCCTGATCCAGTTCCTGC-3’<br>R: 5’-CTGGCATTTGTGGTTGGGTC-3’	hTFE3	F: 5’-GAGCTGCCCAACATCAAACG-3’<br>R: 5’-ATCTCCGGGTCACTGGACT-3’
hIL-10	F:5’- GCTGAGAACCAAGACCCAGAC-3’<br>R:5’- CATTCTTCACCTGCTCCACGG-3’	hTSIX	F:5’-ACAACCACCAATACCAACACAG-3’<br>R: 5’-GCACTATCAGGTAGCAGGCA-3’
hMT-ATP8	F:5’-TACCACCTACCTCCCTCACC-3’<br>R:5’-GGCAATGAATGAAGCGAACAGA-3’	rCOX2	F:5’-TTGCCCAGCACTTCACTCAT-3’<br>R:5’-AGGATACACCTCTCCACCGA-3’
hMT-ND4L	F:5’-ATCGCTCACACCTCATATCCT-3’<br>R:5’-GGAGTGGGTGTTGAGGGTTAT-3’	rGAPDH	F:5’- AGACAGCCGCATCTTCTTGT-3’<br>R:5’-CTTGCCGTGGGTAGAGTCAT-3’
hPKM	F:5’-TGTTTGCGTCATTCATCCGC-3’<br>R:5’-CTAGATCACCACGAGCCACC-3’	rIL-1β	F:5’-GCTACCTATGTCTTGCCCGT-3’<br>R:5’-TCACACACTAGCAGGTCGTC-3’
hPPARγ	F:5’-TGACCAAAGCAAAGGCGAGG-3’<br>R:5’-CCCTGAAAGATGCGGATGGC-3’	rIL-6	F:5’-TCTGCTCTGGTCTTCTGGAGT-3’<br>R:5’-AGGAGAGCATTGGAAGTTGGG-3
hPPARGC1A	F:5’-GCCCAGGTATGACAGCTACG-3’<br>R:5’-CTGTCCGTGTTGTGTCAGGT-3’	rIL-10	F:5’- ACTGCTATGTTGCCTGCTCTT-3’<br>R:5’-TGGCAACCCAAGTAACCCTT-3’
hPPARGC1B	F:5’-GCTCAAGCTCTGGCTCTTCA-3’<br>R:5’-ATGCTTGGCGTTCTGTCTGA-3’	rTNFα	F:5’- ATCGGTCCCAACAAGGAGGA-3’<br>R:5’-CGCTTGGTGGTTTGCTACG-3’

[1] h: human; r: rat

Table S4. Gene expression profile of highly expressed genes in Cluster 4.

Gene	Log_2(FC)*	Gene	Log_2(FC)	Gene	Log_2(FC)	Gene	Log_2(FC)
MT-ND1	2.864	MALAT1	1.483	MMP1	0.934	SLC2A3	0.707
MT-CO3	2.854	GREM1	1.455	THBS2	0.919	ITGA11	0.705
MT-CO1	2.813	COL5A1	1.432	SLC5A3	0.888	APP	0.698
MT-ND3	2.782	MEG8	1.432	POSTN	0.887	LOXL2	0.693
MT-ND4	2.764	COL6A1	1.385	EGR1	0.877	AC016831.5	0.690
MT-CYB	2.737	AC020916.1	1.296	HIF1A-AS3	0.826	NFKBIZ	0.686
MT-CO2	2.576	CARMN	1.294	MMP2	0.825	CTSD	0.668
MT-ND2	2.534	COL1A2	1.283	NR4A2	0.806	ATP13A3	0.662
MT-ATP6	2.529	HMCN1	1.202	GPNMB	0.796	HES1	0.655
MT-ND5	2.500	PXDN	1.181	ADAM12	0.773	NRP2	0.641
MT-ND6	2.183	COL7A1	1.127	ANPEP	0.766	COL5A3	0.633
MT-ND4L	2.178	FN1	1.125	AL138828.1	0.750	ATF3	0.619
NEAT1	1.937	PAPPA	1.125	REV3L	0.740	SAT1	0.615
TSIX	1.907	COL3A1	1.090	HMGA2	0.731	COL4A1	0.608
MEG3	1.893	BX322234.1	1.043	LINC01705	0.722	LMO7	0.607
MT-ATP8	1.718	HMGA2-AS1	0.957	THBS1	0.719	CPD	0.591
COL1A1	1.590	AC037198.1	0.951	TP53INP1	0.716	UACA	0.582

*FC: Fold change
