# Supplementary material for: Enhanced mitochondrial function and delivery from adipose-derived stem cell spheres via the EZH2-H3K27me3-PPARγ pathway for advanced therapy
Source: Stem Cell Res Ther. 2025 Mar 11;16:129. doi: 10.1186/s13287-025-04164-1 (PMC11899936; doi:10.1186/s13287-025-04164-1)
Supplement: Supplementary file 2 — Supplementary Material 2 [file 13287_2025_4164_MOESM2_ESM.pdf]

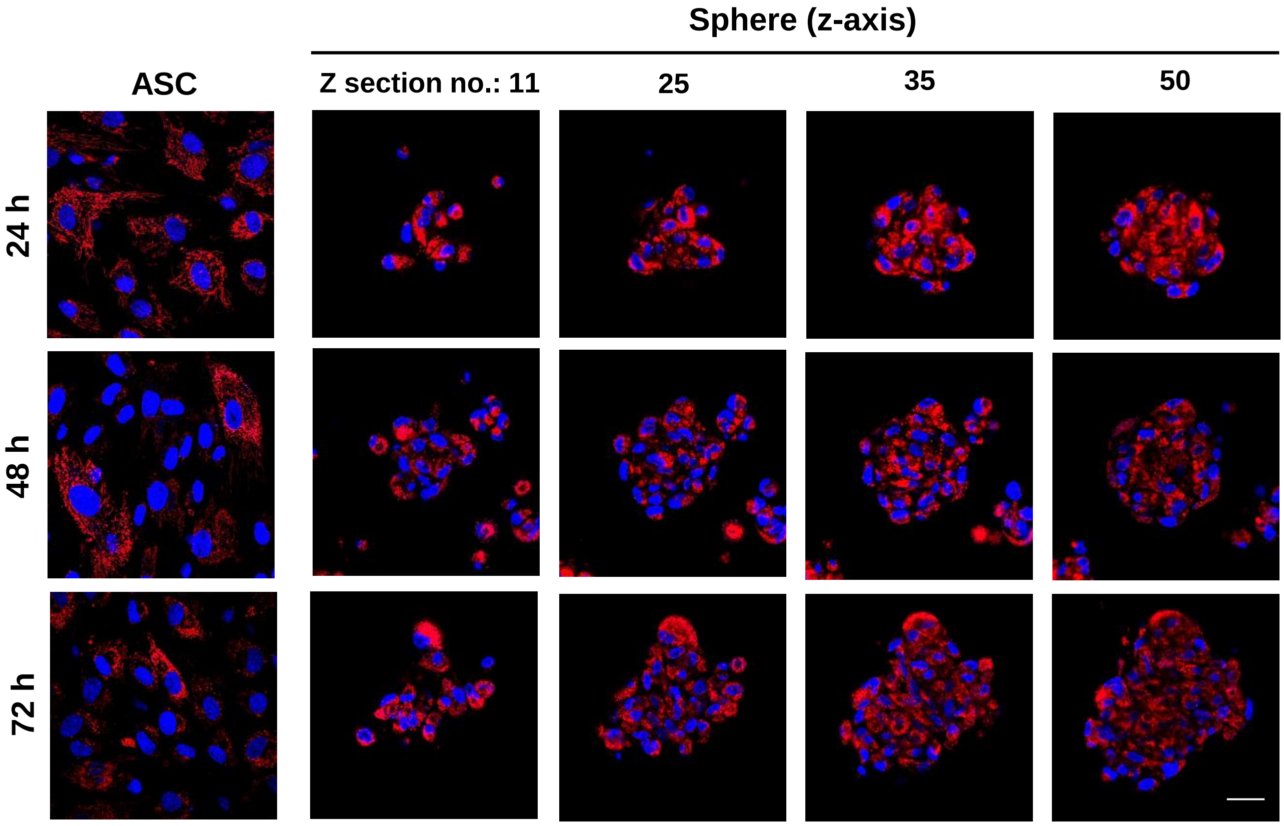

Fig. S1. The morphological changes of mitochondria during the formation of 3D spheres by ASCs. Represenative confocal microscopy images illustrate MitoTracker Deep Red FM-labeled mitochondria in 2D-cultured ASCs and ASC-assembled 3D spheres on chitosan-coated surfaces for 24, 48, and 72 hours. (Scale bar: 20 μm)

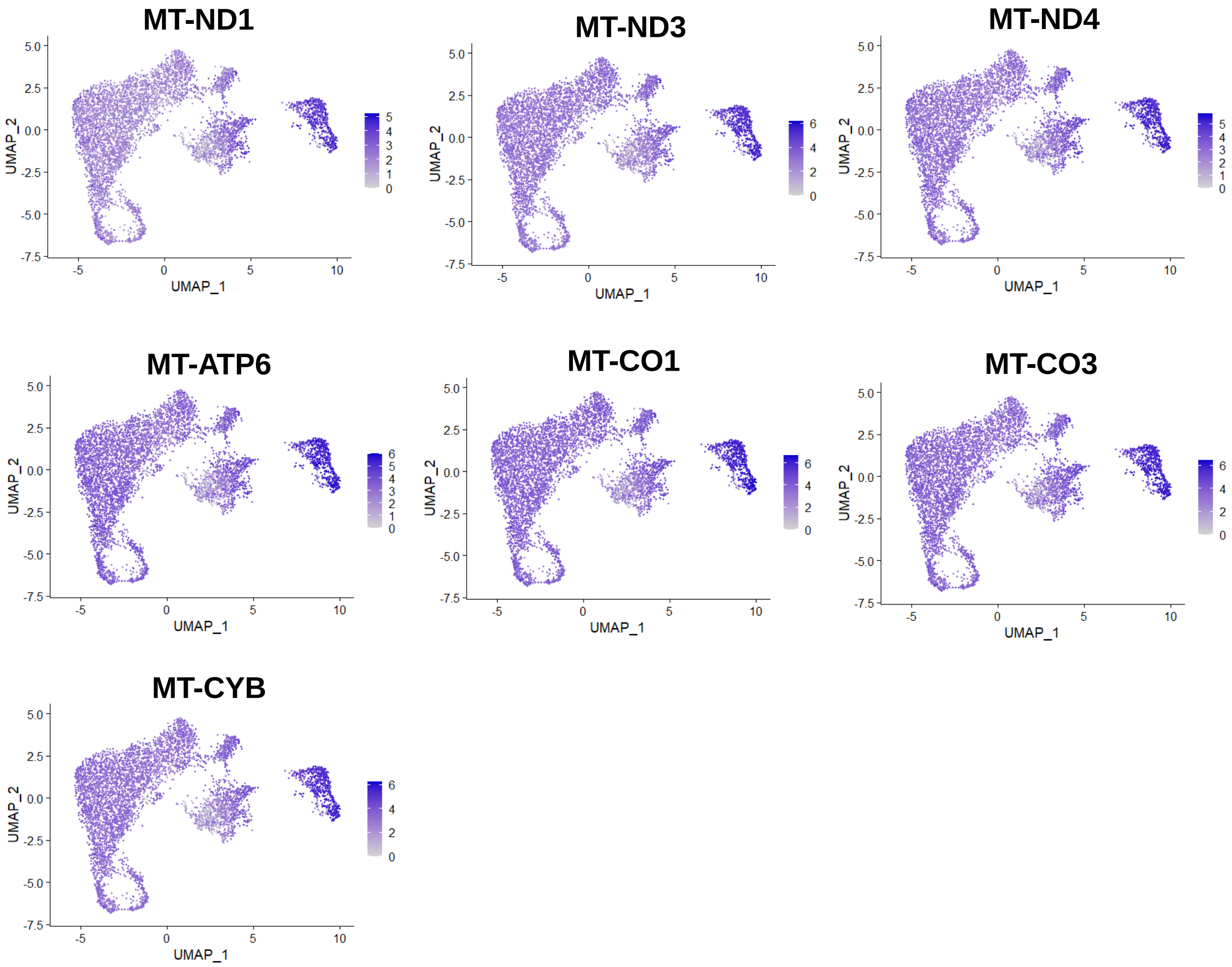

Fig. S2. Expression and distribution map of the top increased genes in Cluster 4.

In addition to the top identified genes shown in Fig. 2C, other highly expressed genes in Cluster 4 during ASC sphere formation are also displayed on the 2D UMAP plot.

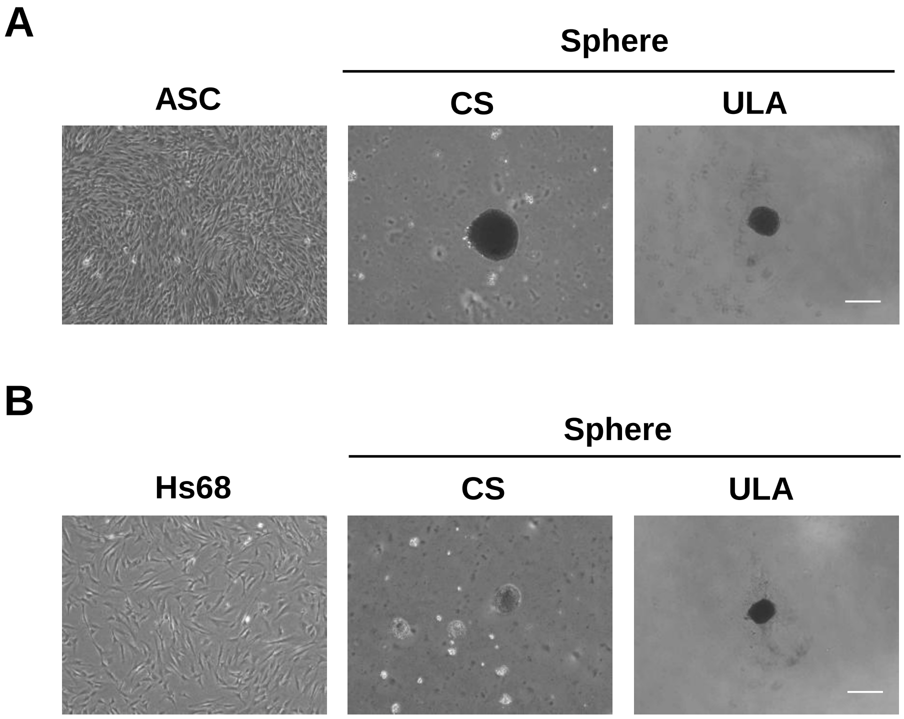

Fig. S3. Sphere formation can be induced by both chitosan-coated surfaces (CS) and ultra-low attachement (ULA) plate in ASCs and Hs68 cells.

Phase contrast images demonstrated successful sphere formation with various sizes and morphologies of **(A)** ASC spheres or **(B)** Hs68 spheres formed on CS or ULA plates. Original magnification: 40x. Scale bar: 200 μm.

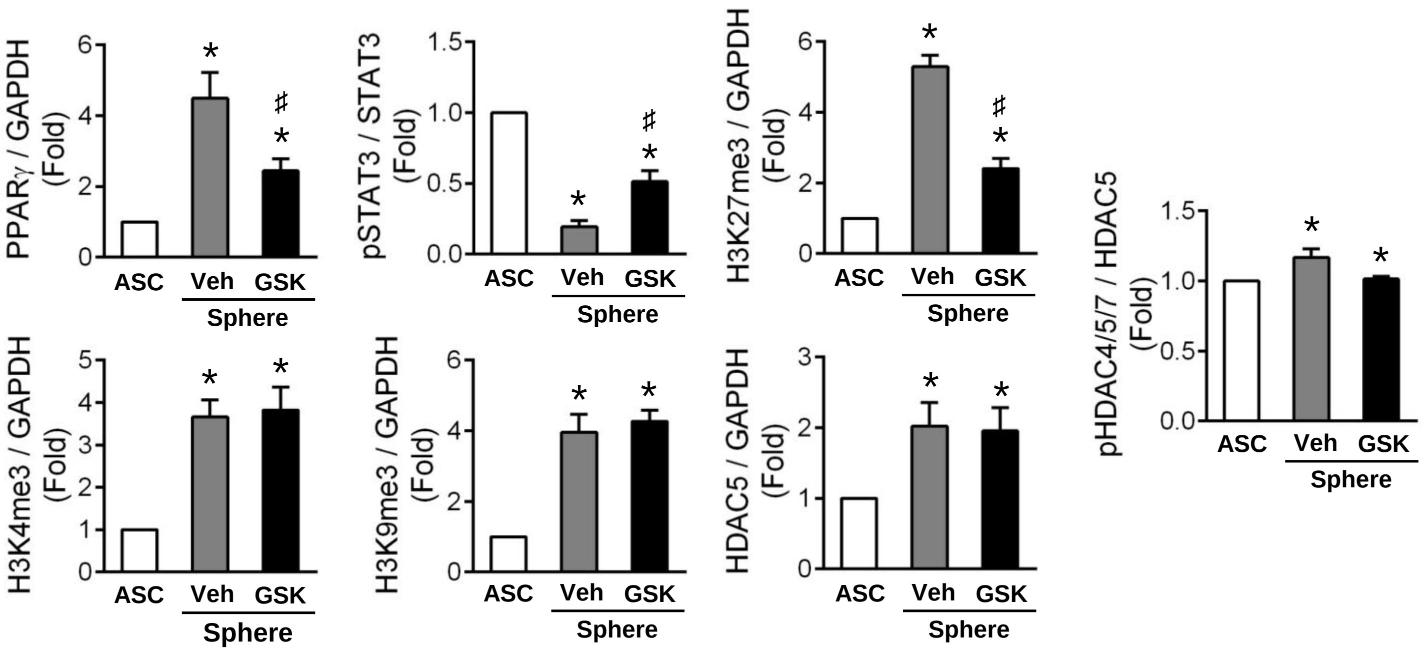

Fig. S4. The quantification and statistical analysis of the western blotting results shown in Figure 5B. The bar chart illustrates the average protein levels (n = 3) of PPARγ, pSTAT3, STAT3, H3K4me3, H3K9me3, H3K27me3, p-HDAC4/5/7, and HDAC5. These levels reflect sphere-induced protein expressions, with a decrease in PPARγ, STAT3, and H3K27me3 following treatment with the GSK inhibitor. GAPDH was used as a loading control. Data were normalized to GAPDH and presented as relative values compared to the ASC group. All values are expressed as mean ± SEM and were analyzed using one-way ANOVA with Tukey's multiple comparisons post-test. *$p < 0.05$ compared to the ASC control group. ♯$p < 0.05$ compared to the Sph Veh group.

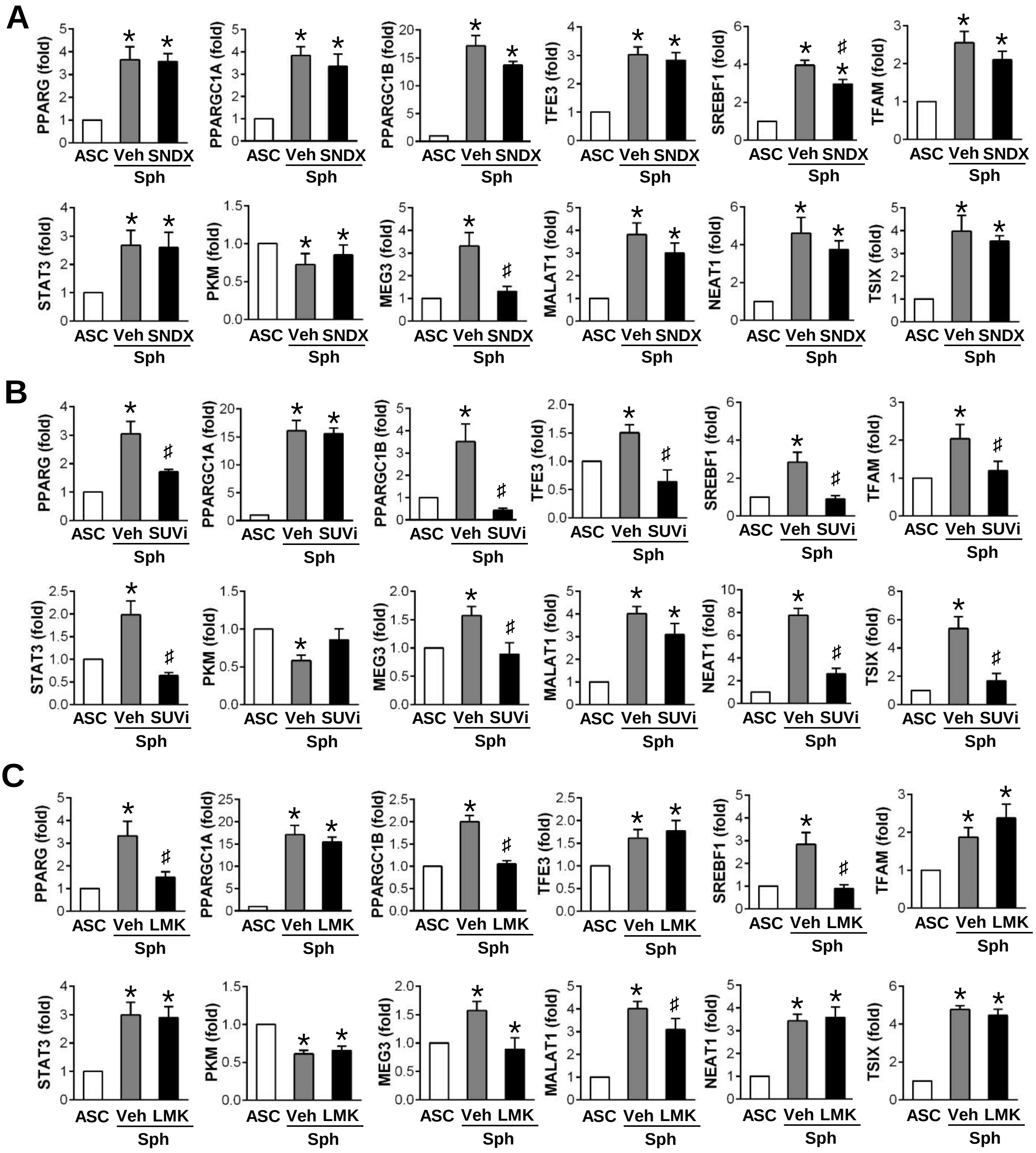

Fig. S5. Partial inhibitions of Cluster 4 and IPA-predicted PPARγ-associated genes by other epigenetic inhibitors.

The qPCR showed some decreases in PPARγ, PPARG1A, PPARG1B, SERBF1, STAT3, TFE3, TFAM, PKM, and Neat1 gene expressions during 3D sphere formation when treated with **(A)** SNDX-5613, **(B)** SUVi, and **(C)** LMK235. Data were analyzed by one-way ANOVA with Tukey's multiple comparisons post-test. * $p<0.05$ vs. the 2D-cultured ASC group. ♯ $p<0.05$ vs. the CS-induced ASC sphere Veh group.

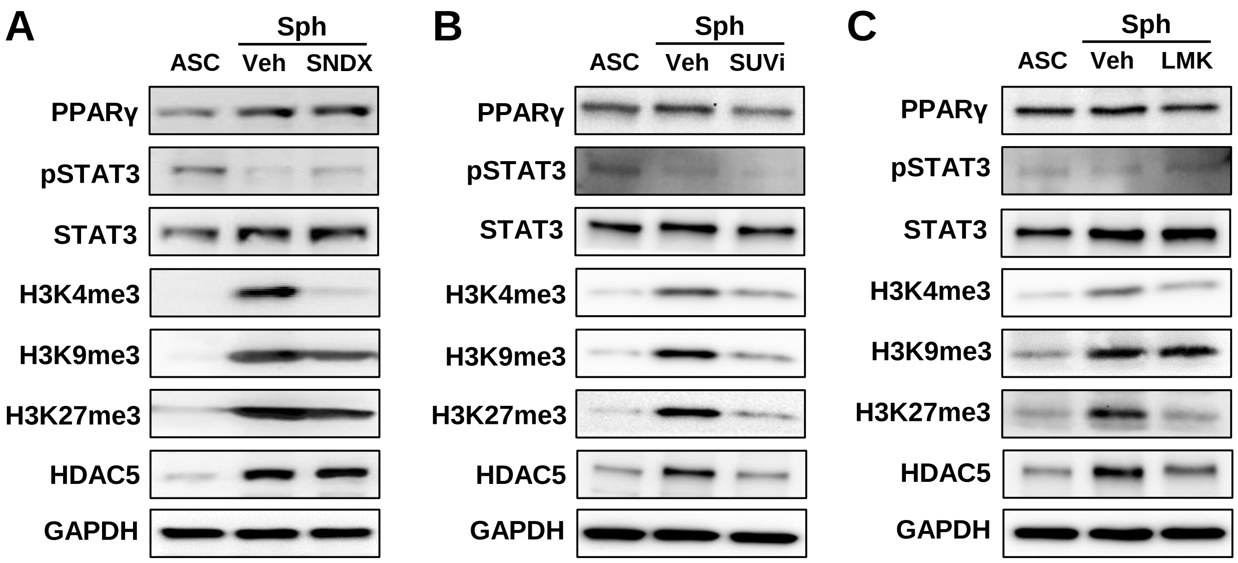

Fig. S6. Profiling protein expressions between 2D-cultured ASCs and 3D ASC spheres with or without other epigenetic inhibitors.

Western blotting analysis of PPARγ, pSTAT3, STAT3, H3K4me3, H3K9me3, H3K27me3, and HDAC5 expressions in ASC spheres treated with **(A)** SNDX-5613, **(B)** SUVi, and **(C)** LMK235 for 72 hours during chitosan-induced sphere formation.

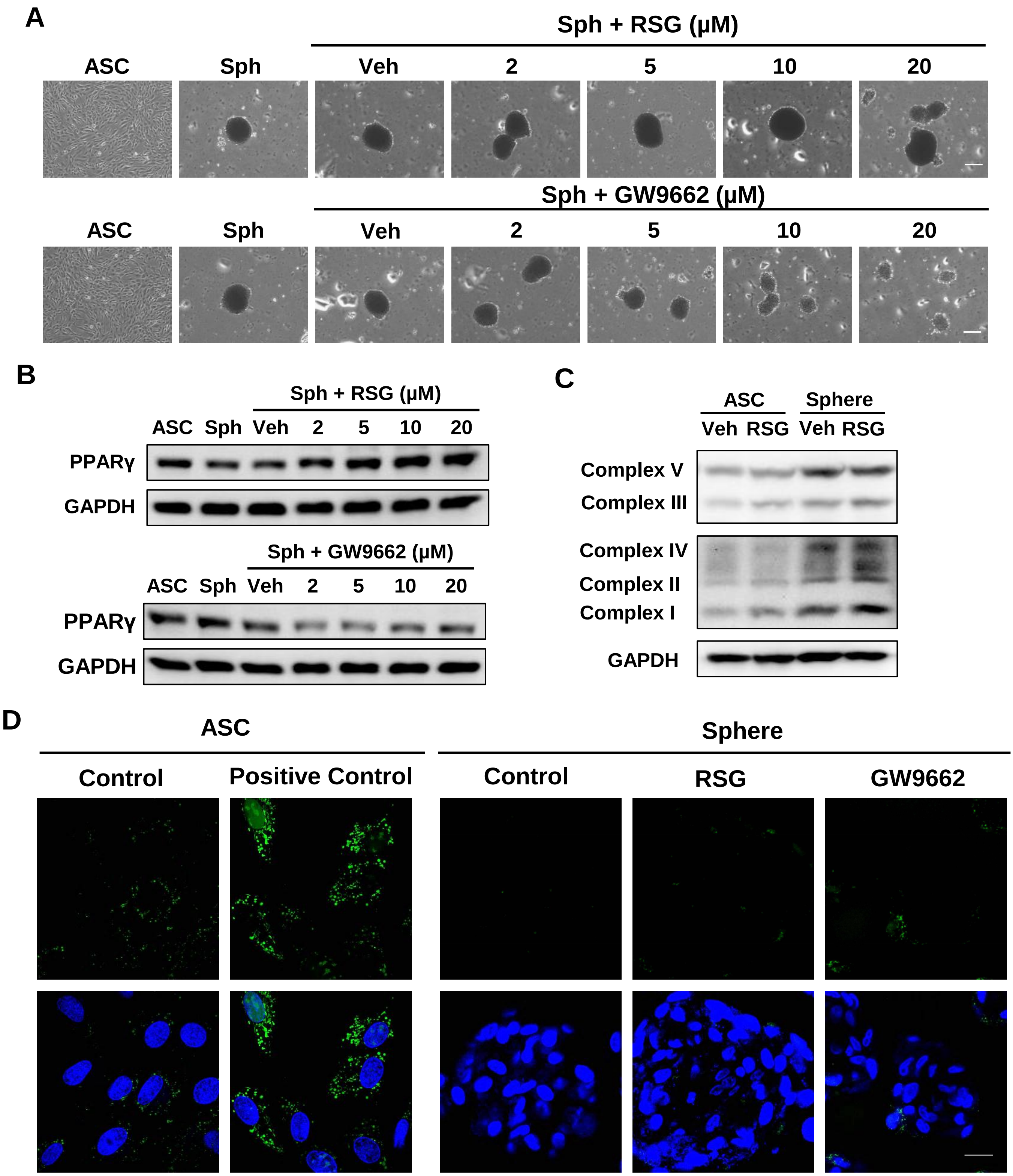

Fig. S7. PPARγ plays a vital role in ASC sphere assembly, influencing sphere size and ROS levels.

(A) Phase contrast images showed size and morphological changes in ASC spheres (Sph) with additional treatments of DMSO (Veh), PPARγ agonist (RSG), and PPARγ antagonist (GW9662). (Scale bar: 200 μm) **(B)** Western blotting analysis of PPARγ protein expressions in ASC spheres treated with different dosage of RSG or GW9662. **(C)** Western blotting analysis of total lysate to illustrate the expressions of mitochondria complex I-V proteins in 2D-cultured ASCs and 3D spheres with or without additional 20 μM RSG treatments. **(D)** Representative images of ROS levels in 2D-cultured ASCs (Control), LPS-induced ASCs (positive control, 100μM LPS), 3D ASC Spheres (Control), Spheres treated with RSG (RSG), and Spheres treated with GW9662 (GW9662). (Scale bar: 20 μm)

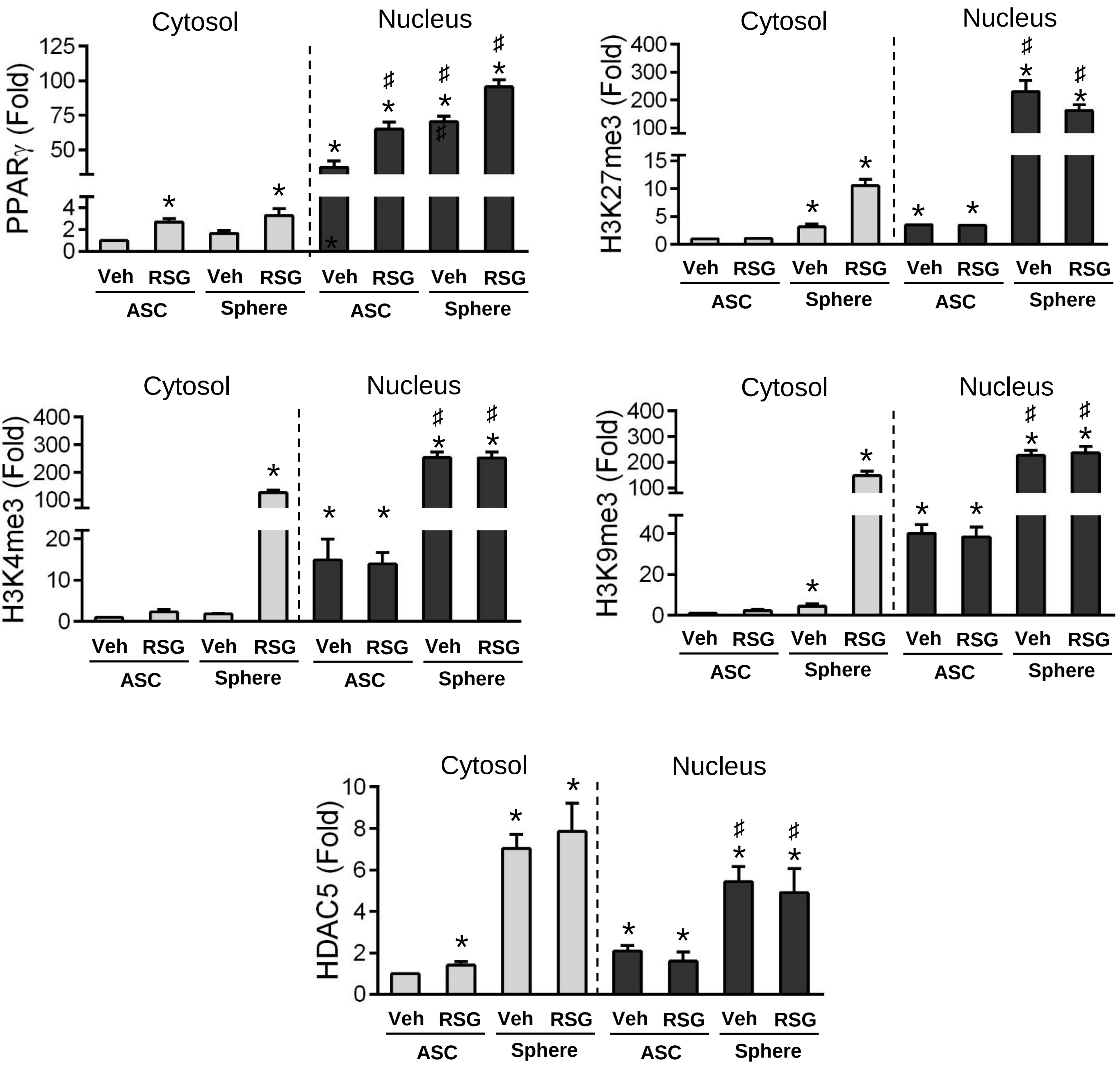

Fig. S8. The quantification and statistical analysis of the western blotting results shown in Figure 5E. The average protein expressions (n=3) of PPARγ, H3K27me3, H3K4me3, H3K9me3, and HDAC5 in the cytosolic and nuclear fractions of 2D-cultured ASCs and 3D ASC spheres, with or without RSG treatment, were analyzed. GAPDH and Lamin A/C served as markers and loading controls for the cytoplasmic and nuclear fractions, respectively. Data were normalized to the respective loading control and presented as relative values compared the cytosolic fraction of the ASC group. All values were expressed as mean ± SEM and analyzed by one-way ANOVA with Tukey's multiple comparisons post-test. *p<0.05 compared to the cytosolic fraction of the ASC group. ASC control group. ♯p<0.05 compared to the nucleus fraction of the ASC group. .

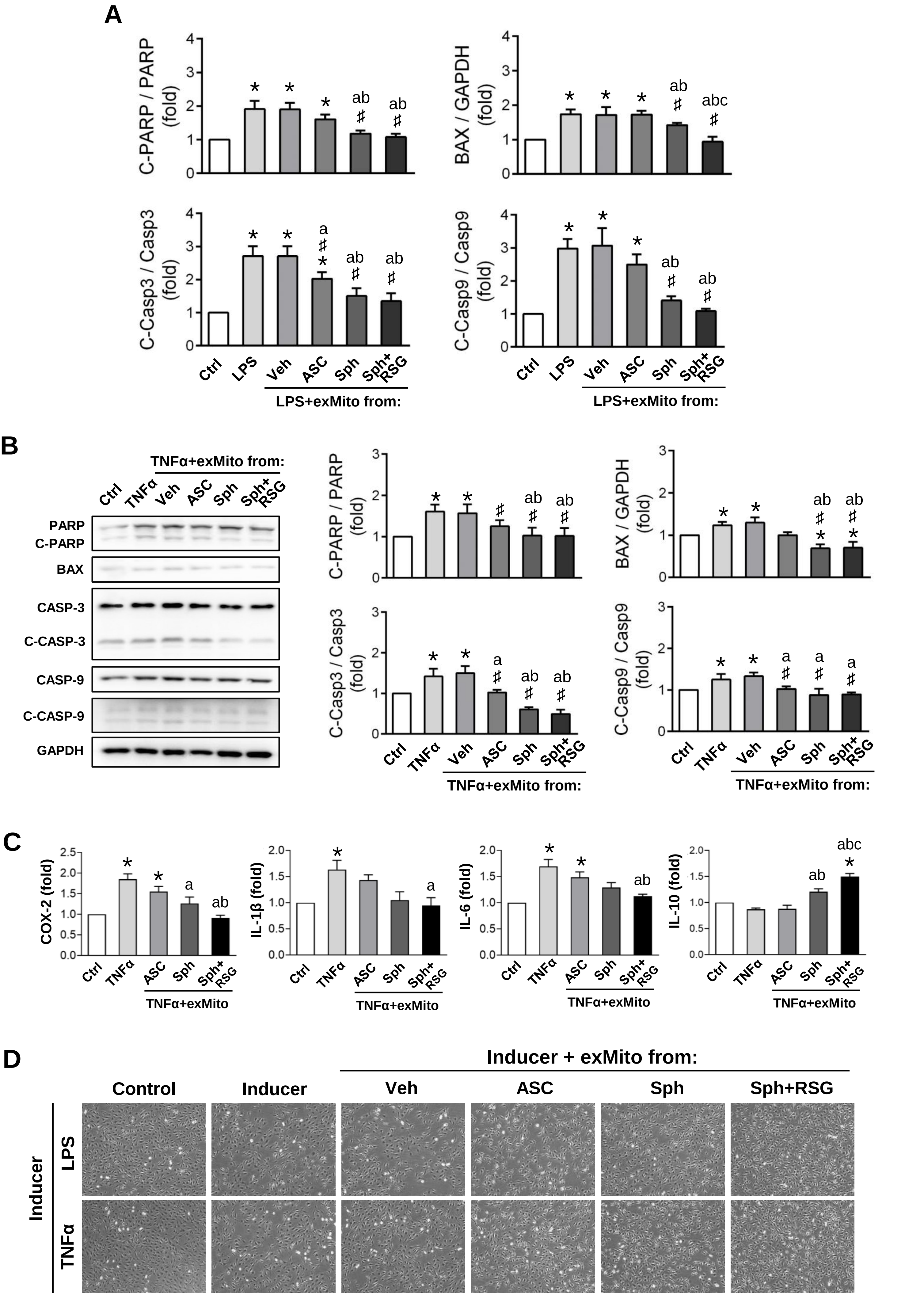

A
C-PARP / PARP (fold)
BAX / GAPDH (fold)
C-Casp3 / Casp3 (fold)
C-Casp9 / Casp9 (fold)
Ctrl
LPS
Veh
ASC
Sph
Sph+ RSG
LPS+exMito from:
B
TNFα+exMito from:
PARP
C-PARP
BAX
CASP-3
C-CASP-3
CASP-9
C-CASP-9
GAPDH
TNFα
C
COX-2 (fold)
IL-1β (fold)
IL-6 (fold)
IL-10 (fold)
TNFα+exMito
D
Inducer + exMito from:
Control
Inducer
Veh
ASC
Sph
Sph+RSG
LPS
TNFα

Fig. S9. PPARγ serves as a key downstream pathway of EZH2, enhancing mitochondrial functions during ASC sphere formation.

(A) The quantification and statistical analysis of the Western blotting results shown in Figure 6B are presented. The bar chart illustrates the average protein levels of PARP, cleaved PARP (C-PARP), Caspase-3 (Casp3), cleaved Caspase-3 (C-Casp3), Caspase-9 (Casp9), and cleaved Caspase-9 (C-Casp9) in RT4 cells treated with 20 μg/ml LPS (LPS group). The extracted mitochondria (exMito) were isolated from 2D-cultured ASCs (ASC), ASC spheres (Sph), or 20 μM RSG-treated 3D ASC spheres (Sph+RSG). These mitochondria were added to the RT4 cells 3 hours after LPS-induced inflammation. Data in were normalized to GAPDH (loading control) and presented as relative values compared to the ASC group. All values are expressed as mean ± SEM (n=3) and were analyzed using one-way ANOVA with Tukey's multiple comparisons post-test. *$p < 0.05$ compared to the ASC control group. ♯$p < 0.05$ compared to the LPS group. $^{a}p < 0.05$ compared to the LPS+Veh group. $^{b}p < 0.05$ compared to the LPS+ASC exMito. $^{c}p < 0.05$ compared to the LPS+Sph exMito group. **(B)** Western blot analysis of TNFα-induced apoptosis markers (listed in panel A) and their reduction following treatments with various extracted mitochondria (exMito) from 2D-cultured ASCs (ASC), 3D spheres (Sph), and 3D spheres with additional RSG (Sph+RSG). The quantification and statistical analysis (right panel) of the Western blotting results are shown in the right panel. Data were normalized to GAPDH (loading control) and presented as relative values compared to the ASC group. All values are expressed as mean ± SEM (n=3) and were analyzed using one-way ANOVA with Tukey's multiple comparisons post-test. *$p < 0.05$ compared to the ASC control group. ♯$p < 0.05$ compared the TNFγ group. $^{a}p < 0.05$ compared to the TNFγ+Veh group. $^{b}p < 0.05$ compared to the TNFγ+ASC exMito group. **(C)** The real-time PCR experiment was conducted to assess the inhibition of TNFα-induced inflammatory cytokine mRNA expression by various exMito treatments. Data in were normalized to GAPDH (loading control) and presented as relative values compared to the ASC group. All values are expressed as mean ± SEM (n=4) and were analyzed using one-way ANOVA with Tukey's multiple comparisons post-test. *$p < 0.05$ compared to the ASC control group. ♯$p < 0.05$ compared to the TNFγ group. $^{a}p < 0.05$ compared to the TNFγ+ASC group . $^{b}p < 0.05$ compared to the TNFγ+ exMito group. $^{c}p < 0.05$ compared to the TNFγ+Sph exMito group. **(D)** Phase contrast images demonstrated changes in cell morphology in RT4 cells after exposure to inflammatory inducers, LPS (20 μg/ml) or TNFα (10 ng/ml), followed by treatment with different exMito (Scale bar: 200 μm).

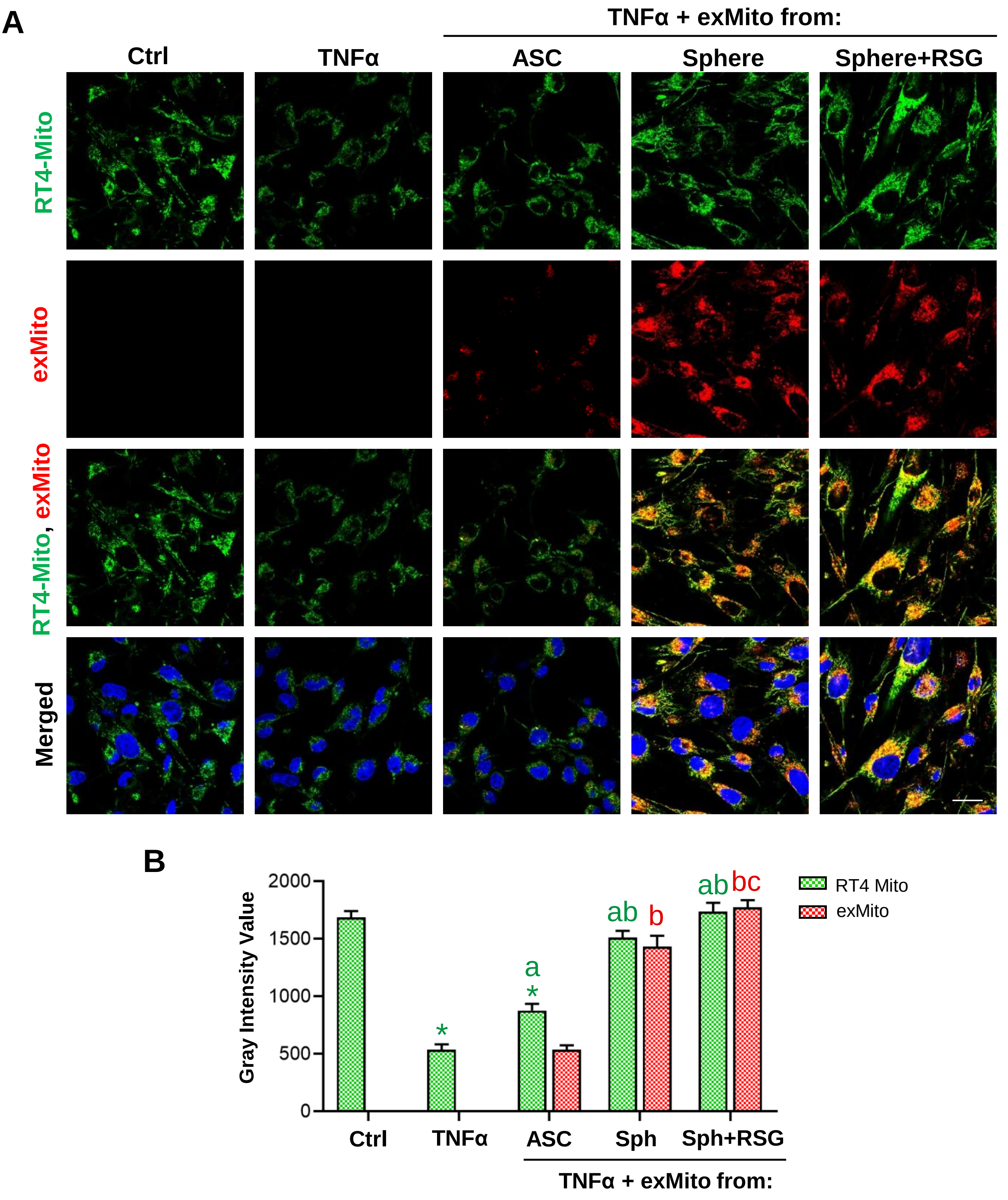

Fig. S10. Evaluation of therapeutic outcomes of extracted mitochondria to rescue TNFα-induced cell damages.

(A) Immunofluorescence images showing mitochondrial morphologies and expressions of both endogenous mitochondria (RT4-Mito, labeled with MitoTracker Green FM dye) and exMT isolated from different ASC groups (exMito, labeled with MitoTracker DeepRed FM dye) in 10 ng/ml TNFα-inflamed RT4 cells (Scale bar: 20 μm). **(B)** Quantification of fluorescent intensities for endogenous mitochondria (green) and exMito (red) in TNFα-inflamed RT4 cells (n=3). All values are represented as the mean ± SEM and analyzed by one-way ANOVA with Tukey's multiple comparisons post-test. *$p<0.05$ compared to the RT4 control group (Ctrl). $^{a}p<0.05$ compared to the TNFα-induced group (TNFα). $^{b}p<0.05$ compared to TNFα + exMito from ASC group. $^{c}p<0.05$ compared to TNFα + exMito from Sph group.
