# Supplementary material for: Enhanced mitochondrial function and delivery from adipose-derived stem cell spheres via the EZH2-H3K27me3-PPARγ pathway for advanced therapy
Source: Stem Cell Res Ther. 2025 Mar 11;16:129. doi: 10.1186/s13287-025-04164-1 (PMC11899936; doi:10.1186/s13287-025-04164-1)
Supplement: Supplementary file 12 — Supplementary Material 12 [file 13287_2025_4164_MOESM12_ESM.pdf]

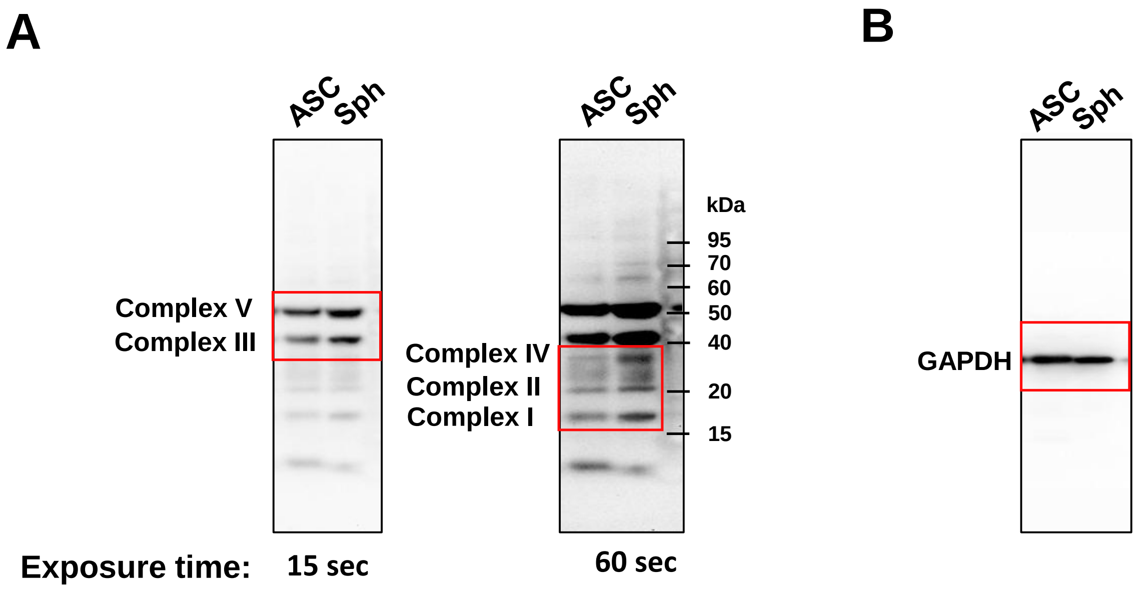

Fig. S11. Uncropped full-length western blots corresponding to Fig. 2D. (A) Mitochondrial electron transport chain complexes and (B) GAPDH. Red boxes indicate the regions that were cropped and shown in the main text.

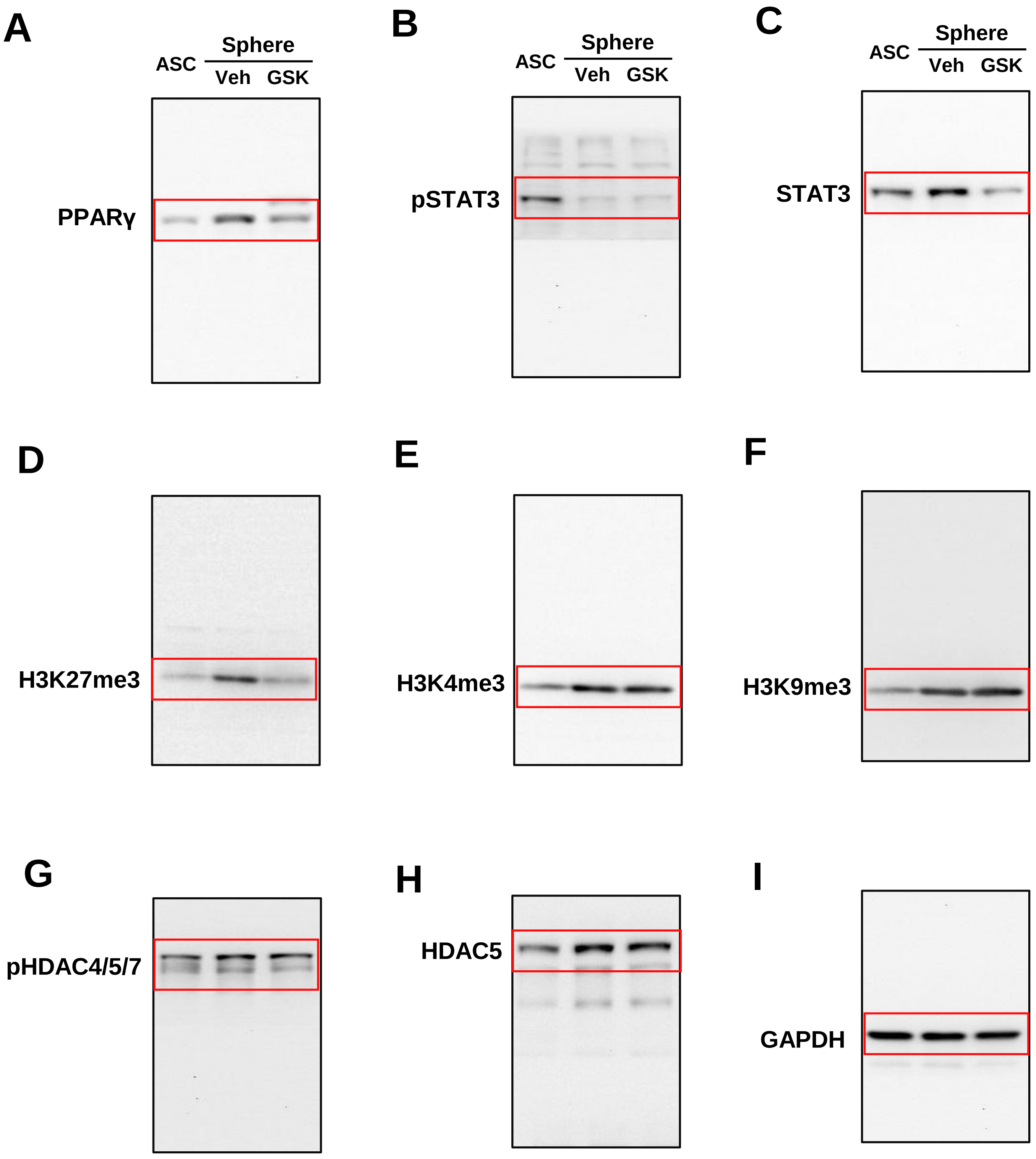

Fig. S12. Uncropped full-length western blots corresponding to Fig. 5B. (A) PPARγ (B) phospho-STAT3, (C) STAT3, (D) H3K27me3, (E) H3K4me3, (F) H3K9me3, (G) phospho-HDAC4/5/7, (H) HDAC5, and (I) GAPDH. Red boxes indicate the regions that were cropped and shown in the main text.

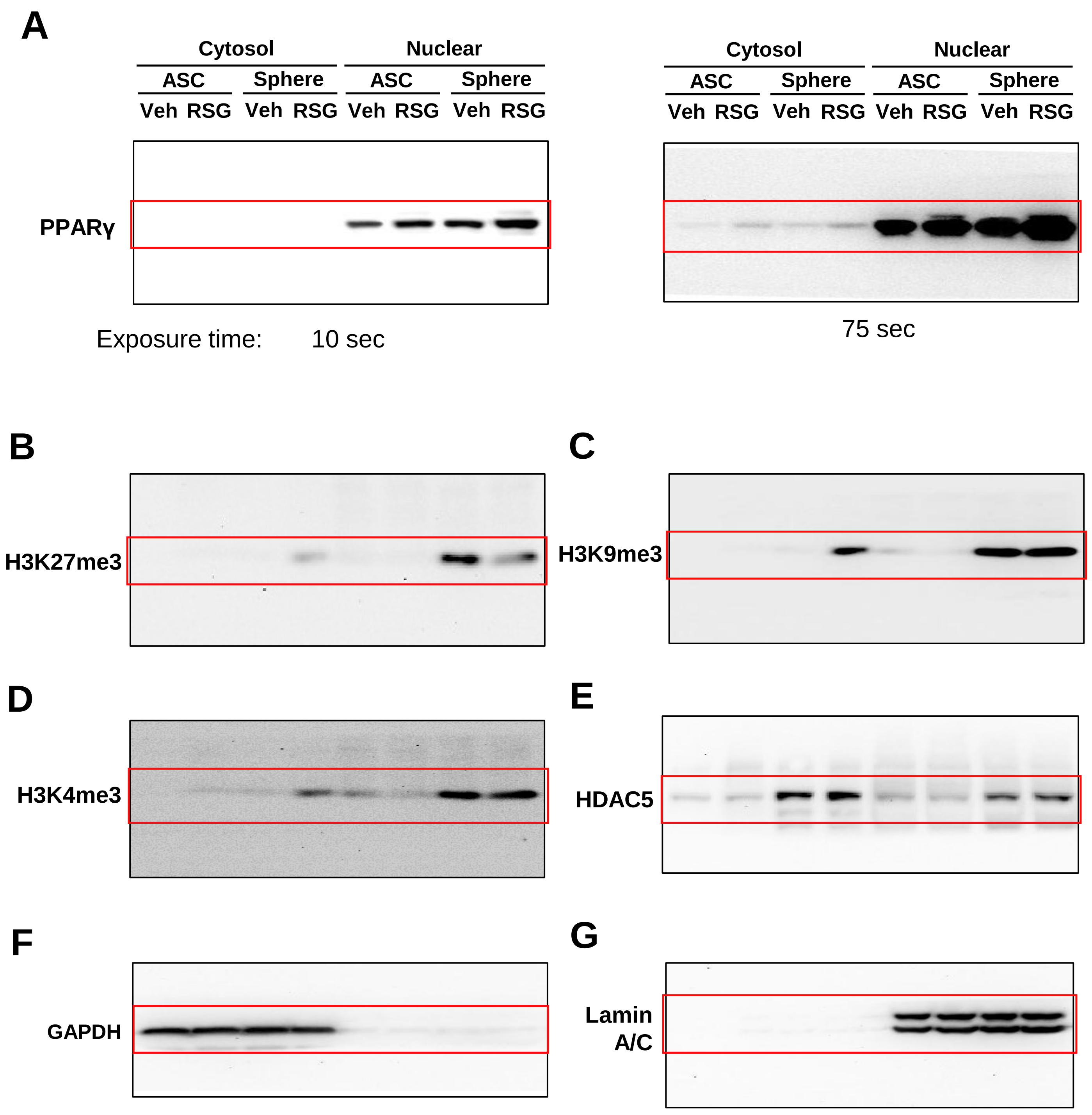

Fig. S13. Uncropped full-length western blots corresponding to Fig. 5E. (A) PPARγ, (B) H3K27me3, (C) H3K9me3, (D) H3K4me3, (E) HDAC5, (F) GAPDH, and (G) Lamin A/C. Red boxes indicate the regions that were cropped and shown in the main text.

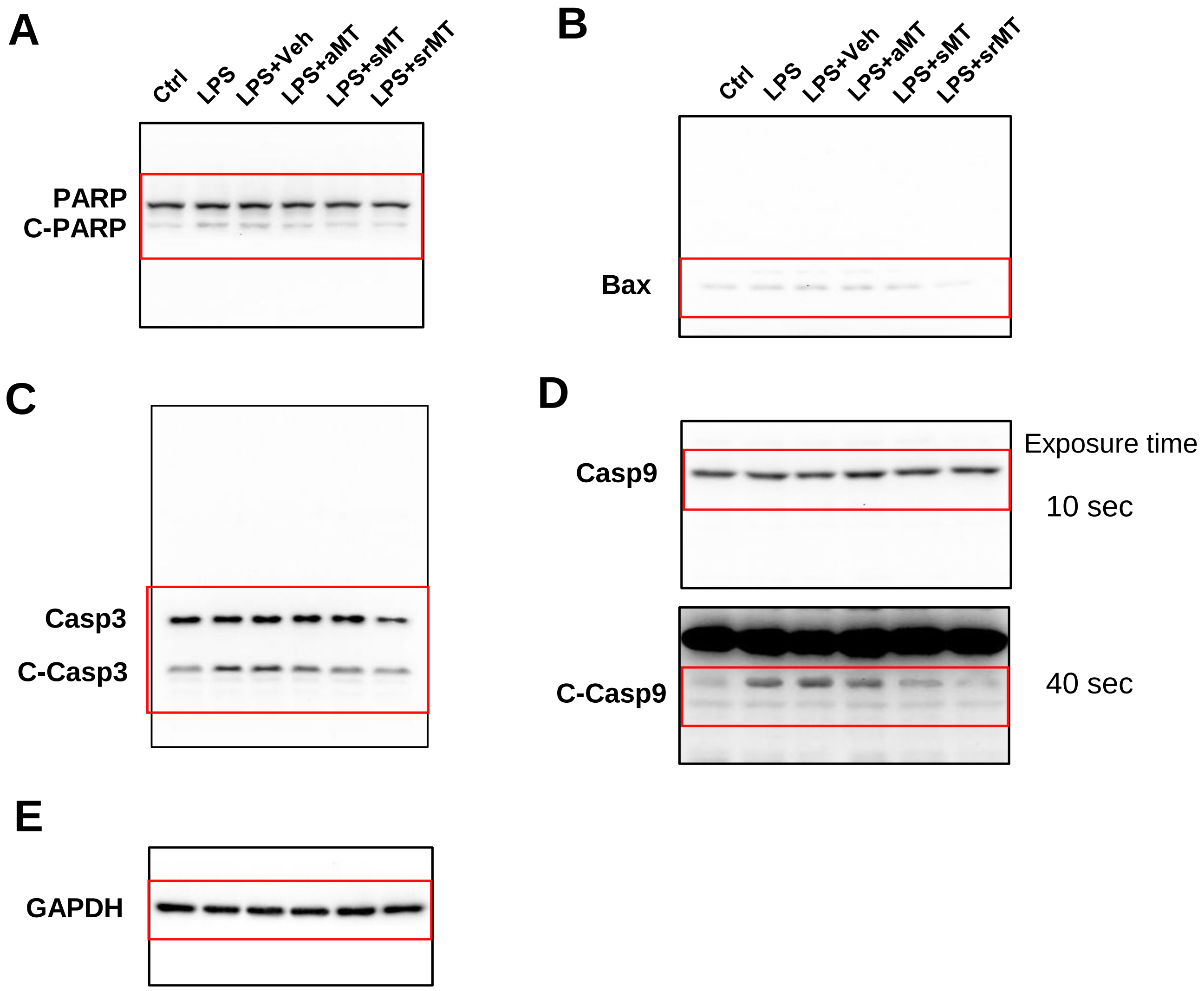

Fig. S14. Uncropped full-length western blots corresponding to Fig. 6B. (A) PARP and cleaved PARP, (B) BAX, (C) Caspase-3 and cleaved Caspase-3, (D) Caspase-9 and cleaved Caspase-9, and (E) GAPDH. Red boxes indicate the regions that were cropped and shown in the main text.
