# Supplementary material for: Enhanced mitochondrial function and delivery from adipose-derived stem cell spheres via the EZH2-H3K27me3-PPARγ pathway for advanced therapy
Source: Stem Cell Res Ther. 2025 Mar 11;16:129. doi: 10.1186/s13287-025-04164-1 (PMC11899936; doi:10.1186/s13287-025-04164-1)
Supplement: Supplementary file 13 — Supplementary Material 13 [file 13287_2025_4164_MOESM13_ESM.pdf]

Documents for Human Cells Used in This Study

1. Product Certificate for human ASCs (C12977, hMSC-AT, PromoCell GmbH)

2. Proof of Purchase for Hs68 cells from BCRC, FIDIC, Hsinchu, Taiwan.

3. Material Transfer Agreement (MTA) for Hs68 cells.

4. Material Transfer Agreement (MTA) for Hs68 cells (English translation).

5. Product Information Sheet for Hs68 cells.

Certificate of Analysis

PromoCell

Human Mesenchymal Stem Cells from Adipose Tissue (hMSC-AT)

Product Description

Product Name	hMSC-AT-c	hMSC-AT-p
Order Number	C-12977	C-12978
Lot Number	476Z008	
Amount per Unit	≥ 500.000 cells in Cryo-SFM (Order No.: C-29910)	≥ 500.000 cells in MSC Growth Medium 2 (Order No.: C-28009)
Condition	Cell solution cryopreserved	Proliferating cell culture
Package Size	1.2 ml Cryo-Vial	T25 cell culture flask
Stage of Culture	thawing and seeding results in passage 2 (3rd culture)	shipped in passage 2 (3rd culture)
Date of Manufacture	08/2021	---
Expiry Date	n/a if stored under defined condition (Instruction Manual)	
QC Evaluation Medium	MSC Growth Medium 2 (Order No.: C-28009)<br>MSC Adipogenic Differentiation Medium 2 (Order No.: C-28016)<br>MSC Chondrogenic Differentiation Medium (Order No.: C-28012)<br>MSC Osteogenic Differentiation Medium (Order No.: C-28013)	

Approved Tissue Donor Program

Donor Information

Donor Age / Sex / Race	46 / female / caucasian
Tissue / Localisation	subcutaneous adipose tissue / abdomen

The tissue used by PromoCell for the isolation of human cell cultures is derived from donors who have signed an informed consent form, which outlines in detail the purpose of the donation and the procedure for processing the tissue (www.promocell.com/ethics).

Results of Analysis

Growth Characteristics	Test Method	Specification	Result
Cell Count	Automated fluorescent live / dead cell staining method (Viacount Assay / Muse Cell Analyzer, Millipore)	≥ 500.000 cells	650.000 cells
Viability		≥ 75 %	84 %
Population Doublings	Test performed by thawing cryopreserved cells and using PromoCell's standardized culture system and procedures. The stated values may vary under customer culture conditions.	≥ 10 PD	pass
Median Population Doubling Time incl. lag phase over 10 PDs		≤ 30 h	23 h
**Identity & Purity**			
	Analysed in passage 3 using a Guava Flow Cytometer (Millipore)		
CD105	Antibody provided by BioLegend No. 323208.	≥ 90 %	98 %
CD73	Antibodies provided by Miltenyi Biotec MSC Phenotyping Kit human No. 130-125-285.	≥ 90 %	100 %
CD90		≥ 90 %	100 %
CD14 / CD19/ CD34 / CD45		≤ 10 %	0 %
HLA-DR	Antibody provided by BioLegend No. 307604.	≤ 10 %	0 %
**Potency**			
Adipogenesis	Sudan III staining performed with P3 or P4 cells on day 12-14	pass	pass
Chondrogenesis	Alcian Blue staining performed with P3 or P4 cells on day 21-23	pass	pass
Osteogenesis	Alizarin Red S staining performed with P3 or P4 cells on day 12-14	pass	pass
**Sterility and Virus Testing**			
Bacteria, Fungi	Growth Promotion Test	negative	negative
Mycoplasma Genus, *Mycoplasma pulmonis*	PCR	negative	negative
HIV-1, HIV-2	PCR	negative	negative
HBV, HCV	PCR	negative	negative
HTLV-1, HTLV-2	PCR	negative	negative

See MSC Analysis Application Notes for reference protocols: http://www.promocell.com/application-notes

Uta Boss

Uta Boss, Quality Assurance

Date: Feb 02, 2022

FOR IN VITRO RESEARCH USE ONLY. NOT FOR DIAGNOSTIC OR THERAPEUTIC PROCEDURES.

PromoCell GmbH
Sickingenstr. 63/65
69126 Heidelberg
Germany

North America 1 866 251 2860 (toll free)
Deutschland 0 800 776 6623 (gebührenfrei)
France 0 800 909 332 (ligne verte)
United Kingdom 0 800 960 333 (toll free)
Other Countries + 49 6221 649 340

Email: info@promocell.com
www.promocell.com

Food Industry Research and Development Institute
Bioresource Collection and Research Center
331, Shih-Pin Road, Hsinchu 300, Taiwan
http://www.bcrc.firdi.org.tw
Tel: +886-3-5223191~6 Fax: +886-3-5224172

Proof of Purchase

The following strains have been purchased by Chia-Ching Wu on July 05, 2023.

Strain name	BCRC Number	Lot No.
Hs68	60038	02631

Note:

1. The above information has been confirmed as correct to open the proof.
2. The buyer shall store, handle, and use the biological material in accordance with applicable laws.
3. This proof of purchase is for record use only.

Applicant: Chia-Ching Wu
Address: Department of Cell Biology and Anatomy, College of Medicine, National Cheng Kung University, Tainan, Taiwan., No.1, University Road, Tainan City 70101, Taiwan (R.O.C)
Tel: +886-6-2353535 ext. 5327
Fax: +886-6-2093007

FOOD INDUSTRY RESEARCH & DEVELOPMENT INSTITUTE
財團法人食品工業發展研究所

Food Industry Research & Development Institute
Date: October 11, 2024

Notice Regarding the Purchased Strains

1. Definition

(1) Biological Material: the material supplied to the applicant by FIRDI as identified on the above item II, and it's unmodified progeny (such as replicate from the material), and it's unmodified derivatives (including unmodified functional subunits or products, such as, purified or fractionated subsets of the material, including expressed proteins or extracted or amplified DNA/RNA)

(2) Modifications: substances produced by the applicant by using the Biological Material, which are not the Biological Material, and which have new properties.

(3) Commercial Use: the sale, lease, exchange, license or other use of the Biological Material for financial gain or other for-profit purposes, including but not limited to use of the Biological Material (a) to provide a service to any third party; or (b) to produce or manufacture products for sale.

(4) Applicant: the person as identified on the above item I to be received the Biological Material from FIRDI. For organizational applicant, Applicant includes employees of the organization. For individual applicant supervising a laboratory, Applicant includes people supervised by him/her in the laboratory.

2. Applicant understands and agrees to the following terms and conditions:

(1) **Scope of use: Applicant may use the Biological Material only for research purposes other than Commercial Use. Any Commercial Use of the Biological Material or Modifications is prohibited without FIRDI's prior written consent.**

(2) Caution as to hazards: The Biological Material is experimental in nature and may be hazardous under certain conditions. Especially, the Biological Material designated as biosafety level 2 or above constitutes known pathogen and may cause human diseases. Applicant shall handle and use the Biological Material in accordance with the proper standard of care, so as to avoid harm to persons or the environment.

(3) Caution as to infringement: Applicant shall handle and use the Biological Material with proper attention to avoiding infringement of the intellectual property rights, and other rights, of other persons. In any event that the Applicant stores, handles, or uses the Biological Material, or by any other action or conduct with the Biological Material invades or injures the rights of any other person, or his own rights, then the Applicant shall bear responsibility and legal liability for such invasion or injury, without recourse against FIRDI. As between the Applicant and FIRDI, Applicant shall be responsible for any damages, losses, or expenses including attorney fees as may result from Applicant's possession, handling, and use of the Biological Material, and shall not assert against FIRDI any claim related to such damages, losses, or expenses.

(4) Intellectual properties: Applicant understands that there is no licensing or transfer of any intellectual property right or ownership along with the transfer of the Biological Material. The Biological Material supplied by FIRDI is provided as-is, without representations or warranties of non-infringement of intellectual property rights or any other rights. The Applicant agrees to grant a license for any intellectual property rights created through the use of the Biological Material to FIRDI for purposes of conducting its general activities including supply of the Biological Materials, identification, research, and other services.

(5) Compliance with applicable laws: Applicant shall store, handle, and use the Biological Material in accordance with applicable laws, including but not limited to laws respecting transport, import and export, disposal, and biosafety.

(6) Acknowledgment of source in statements and publications: In any statement, declaration or publication that the Applicant may make relating to the Biological Material or the Modifications, including any patent application irrespective whether it be published, applicant shall acknowledge BCRC as the source of such Biological Material and shall identify the Biological Material by its accession number as listed by BCRC.

(7) Prohibition of transfer to any third party: Applicant shall not under any circumstance transfer any of the Biological Material to any third party.

(8) Human use prohibited: The Biological Material supplied by FIRDI shall not be used in human, such as use in human testing, or transplantation.

(9) Qualification of Applicant: FIRDI may refuse to supply the Biological Materials to the Applicant, if FIRDI determines the Applicant's lack of related knowledge, or qualified capacity to ensure biosafety.

(10) Warranty: FIRDI endeavors to assure that the Biological Material supplied by FIRDI being viable and pure. If the Biological Material received by the Applicant is found to be non-viable or not pure, the Applicant shall notify FIRDI by written within the designated period; FIRDI will replace the Biological Material supplied by FIRDI, or provide other remedy within the value no greater than the amount paid by the Applicant for this application. Except as expressly provided above, FIRDI makes no guarantee as to the Biological Material (such as its safety, fitness) or its information (such as the accuracy of scientific name and other related information) in any manner whatever.

(11) Special requirement: All terms and conditions for the Biological Material as specified in the "special requirement" in BCRC catalog or product sheets shall be included in this Agreement, and shall supersede any inconsistent terms and conditions in this Agreement.

財團法人食品工業發展研究所
生物資源保存及研究中心
地址：新竹市食品路331號　信箱：bcrcweb@firdi.org.tw
電話：03-5223191~6 #248, 509　傳真：03-5224172 (優先) 或 03-5224171

生物材料分讓申請書（訂購單）

付款方式： 超商代收
訂單編號： SO202307016
申請類別： 實驗室
客戶編號： FBC029380
機構 / 部門 / 系所名稱： 國立成功大學 細胞生物及解剖學研究所 吳佳慶實驗室
實驗室生物安全等級： BSL2
代表人： 吳佳慶
聯絡人： 林育辰
連絡電話： 0988262043
發票資料：

統一編號	69115908	發票抬頭	國立成功大學
姓名	林育辰	電子信箱	bts89917@gmail.com
電話	0988262043		
寄送地址	701401 台南市東區大學路1號 國立成功大學 成杏校區 醫學院5樓 國立成功大學醫學院 細胞生物與解剖所 吳佳慶老師實驗室		

生物材料寄送資料：

收件人	林育辰	行動電話	0988262043
電話	0988262043	電子信箱	bts89917@gmail.com
寄件地址	701401 台南市東區大學路1號 國立成功大學 成杏校區 醫學院5樓 國立成功大學 細胞生物與解剖所 吳佳慶老師實驗室		
其他說明			

訂購資料：

BCRC編號	生物材料名稱	BSL	管制性	MOST-NCFB	提供型式	單價	數量
60038	Hs68	1	No	No	Frozen Vial	6000	1
						總數量	1
						處理費	700
						總金額	6700

說明一、各類生物材料皆已含稅金。
說明二、BSL（生物安全等級）標示為2時，請加附「生物安全會同意書」。
說明三、訂購細胞材料時，每件訂單需加收處理費700元。
說明四、訂購其他類生物材料時，每件訂單需加收處理費100元。如訂購內容含低溫冷凍管產品時，需另加收處理費700元。
說明五、一次購買二十株（含）以上八五折優待；購買六十株（含）以上八折優待；購買一百株（含）以上七五折優待。惟特殊計畫之服務供應材料無優惠折扣，例如屬於NCFB計畫之微生物或細胞株。
說明六、人類疾病誘導型多潛能幹細胞服務聯盟(iPSC)所屬細胞株之費用請參考訂購單的繳費流程繳費；此類細胞株處理費已由計畫支付。

申請人簽章：

申請人於訂購前已審閱同意書，並同意遵守同意書約定；且申請人了解並保證下列事項與責任：
一、資料提供：申請人保證申請書所載資料之完整性與正確性。
二、免責保證：申請人如因其對生物材料之使用、處理、保存或其他行為致對造成自己或第三人之權益造成損害時，應自行負擔全部責任，申請人不得對食品所主張任何權利；且申請人對食品所因此所生之一切損害及費用（包括合理之律師費用），應負賠償責任。
三、申請人同意機構內之人員違反同意書或規定時，視為申請人違反；以實驗室負責人申請者，同意實驗室內之人員違反同意書或規定時，視為申請人違反。

申請人： 吳佳慶

實驗室負責人分讓訂購，請實驗室負責人本人於申請人處簽章。

2023 年 07 月 05 日

第 1 份 / 共 1 份

20230705123015　T-PS-M041

財團法人食品工業發展研究所生物資源保存及研究中心
生物材料分讓申請同意書

一、定義

本同意書所稱之「生物材料」係指自食品所分讓取得如申請書(訂購單)所載之樣品，及其未經實質修飾之子代（例如，增殖培養之複製物）及其未經實質修飾之衍生物（指包含有未經實質修飾之功能單位或產物，例如，純化物或部分純化之區分物，如經由表現而得之蛋白質或由萃取或放大而得之DNA/RNA）。

本同意書所稱「修飾物」係指生物材料以外，經實質修飾生物材料而產生新性質者。本同意書所稱之「商業使用」係指將生物材料以販賣、租賃、交換、授權或其他以獲利為目的的使用，包括但不限於用以建立商業服務系統、生產商品。

本同意書所稱之「申請人」若為機構則涵蓋機構內之人員，若為實驗室負責人身分之個人則涵蓋所負責實驗室內之人員。

二、申請人瞭解並同意遵守下列約定：

(一)、商業使用注意：生物材料可能存在食品所以外之相關權利人，生物材料或其修飾物若涉及「商業使用」之使用方式，受分讓人應事先另向該相關權利人取得必要之同意後始得為之。

(二)、危害注意：生物材料本質上為實驗用，不排除有危害之可能。特別是，於訂購資料之BSL欄位標示為2或以上之生物材料或其修飾物，可能造成人體疾病。申請人應注意操作安全以避免對人或環境造成危害。

(三)、侵權注意：生物材料之使用不得侵害他人之權利。受分讓人如因其對生物材料之使用、處理、保存或其他行為致對自己或第三人之權益造成損害時，應自行負擔全部責任，受分讓人不得對食品所主張任何權利；且受分讓人對本所因此所生之一切損害及費用，應負賠償責任。

(四)、智慧財產：生物材料之分讓並未涵蓋其智慧財產權或所有權之授權或移轉，且食品所不保證其使用不侵害他人之智慧財產權或其他權利。申請人同意將因使用生物材料所衍生之智慧財產，授權本所使用於生物材料分讓、鑑定、學術研究與服務等一般業務之執行。

(五)、法規配合：申請人就生物材料之使用應符合相關法規之規定，包括運送、進出口、丟棄、生物安全、實驗操作規範等規定。

(六)、發表記載：申請人於進行任何與生物材料或其修飾物相關之發表(含專利申請)時，應載明生物材料分讓自食品所生物資源保存及研究中心，並依據生物資源保存及研究中心目錄資料載明其提供來源及編號。

(七)、轉提供禁止：生物材料不得以任何形式轉提供予第三人。

(八)、人體試驗禁止：生物材料不得作為以人體試驗為目的之利用。

(九)、操作資格：申請人了解並同意，申請人之專業知識與生物安全相關設備與操作能力，經食品所評估不適合者，食品所得拒絕分讓。

(十)、食品所責任限制：食品所致力於提供存活且無汙染之生物材料樣品。申請人所接獲之生物材料樣品若有不存活或污染之狀況，應於指定期限內以書面通知食品所，由食品所進行樣品更換或其他補償措施(以生物材料之訂購金額為上限)。除此之外，由於食品所生物資源保存及研究中心目錄或產品明細中有關生物材料學名、性質及用途之資料，係由寄存者等提供者所提供，食品所對於生物材料(例如生物材料之安全性、適用性)或其資料(例如資料之正確性)不負任何保證責任。

(十一)、特別條款：各別生物材料目錄及產品說明書所載之「特殊要求」內容視為本同意書之一部分，若兩者有牴觸時，以「特殊要求」內容為準。

(十二)、其他約定事項：

Biological Material Transfer Application Form

Payment Method: Convenience Store Payment
Order Number: SO202307016
Application Type: Laboratory
Customer ID: FBC029380
Institution/Department/Institute Name: Chia-Ching Wu's Laboratory, Dept. of Cell Biology and Anatomy, National Cheng Kung University
Laboratory Biosafety Level: BSL2
Representative: Chia-Ching Wu
Contact Person: Yu-Chen Lin
Contact Phone: 0988262043

Invoice Information:

Unified Business Invoice No.:	69115908	Invoice Title:	National Cheng Kung University
Name:	Yu-Chen Lin	Email:	bts89917@gmail.com
Phone:	0988262043		
Shipping Address:	701401, 5th Floor, College of Medicine, National Cheng Kung University, Chengxing Campus, 1 University Road, East District, Tainan City, Taiwan<br>Wu, Chia-Ching's Laboratory, Dept. of Cell Biology and Anatomy, College of Medicine, National Cheng Kung University		

Biomaterial Shipping Information:

Name:	Yu-Chen Lin	Mobile Phone:	0988262043
Phone:	0988262043	Email:	bts89917@gmail.com
Shipping Address:	Chia-Ching Wu's Laboratory, Dept. of Cell Biology and Anatomy, College of Medicine, National Cheng Kung University<br>5th Floor, No. 1 University Road, East District, Tainan City, 701401, Taiwan.		
Additional Information:			

Order Information:

BCRC Number:	Biomaterial Name:	BSL (Biosafety Level):	Controlled:	MOST:	Provision Format:	Unit Price:	Quantity:
60038	Hs68	1	No	No	Frozen Vial	6000	1
						Total Quantity:	1
						Handling Fee:	700
						Total Amount:	6700

Note 1: All types of biomaterials include taxes.
Note 2: When the BSL (Biosafety Level) is indicated as 2, please attach the "Biosafety Committee Consent Form."
Note 3: When ordering cell materials, a handling fee of 700 NTD will be charged for each order.
Note 4: When ordering other types of biomaterials, a handling fee of 100 NTD will be charged per order. If the order includes frozen vial products, an additional handling fee of 700 NTD will be charged.
Note 5: A 15% discount is offered when purchasing 20 or more strains; a 20% discount for 60 or more strains; and a 25% discount for 100 or more strains. However, there is no discount for materials provided under special service programs, such as microorganisms or cell lines belonging to the NCFB project.
Note 6: For costs related to cell lines under the Human Disease Induced Pluripotent Stem Cells Service Alliance (iPSC), please refer to the payment process on the order form. Handling fees for such cell lines are already covered by the project.

Applicant's Signature:

The applicant has reviewed the consent form prior to placing the order and agrees to comply with the terms of the consent form. The applicant understands and guarantees the following matters and responsibilities:
1.Data Provision: The applicant guarantees the completeness and accuracy of the information provided in the application form.
2.Disclaimer of Liability: If the applicant causes damage to themselves or third parties due to the use, handling, storage, or other actions regarding the biomaterials, the applicant shall bear all responsibilities and shall not claim any rights against the biomaterials. The applicant shall be liable for all damages and costs incurred (including reasonable attorney fees) as a result of such claims.
3.Agreement Compliance: The applicant agrees that any violation of the consent form or regulations by personnel within the institution shall be regarded as a violation by the applicant. In the case where the laboratory head submits the application, any violation of the consent form or regulations by personnel in the laboratory shall also be regarded as a violation by the applicant.

Applicant:

The laboratory head is transferring the order; please have the laboratory head sign at the applicant's section.

Food Industry Research and Development Institute
Bioresource Collection and Research Center

Biological Material Transfer Agreement

I. Definitions

In this agreement, "Biological Material" refers to the samples obtained through the transfer from the Institute of Food Science as described in the application form (order form), including their unmodified progeny (e.g., proliferated copies) and their unmodified derivatives (i.e., containing unmodified functional units or products, such as purified or partially purified fractions, e.g., proteins expressed or DNA/RNA extracted or amplified).

"Modified Materials" refer to those materials, other than Biological Materials, that have acquired new properties through substantial modification of Biological Materials.

"Commercial Use" refers to the use of Biological Materials for profit, including but not limited to sale, lease, exchange, licensing, or other uses aimed at generating profit, including establishing commercial services or producing products.

"Applicant" refers to the individual or organization requesting the materials. If the applicant is an institution, it covers the personnel within that institution. If the applicant is a laboratory head, it covers the personnel within that laboratory.

II. The applicant acknowledges and agrees to abide by the following terms:

1. Commercial Use Note: Biological Materials may be subject to rights held by parties other than the Institute of Food Science. If the Biological Materials or their Modified Materials are intended for Commercial Use, the recipient must first obtain the necessary consent from the relevant rights holders.
2. Hazard Note: Biological Materials are intended for experimental use and may pose hazards. Especially materials marked as BSL-2 or higher in the order details may cause human diseases. The applicant should take safety precautions to avoid harm to people or the environment.
3. Infringement Note: The use of Biological Materials should not infringe on the rights of others. The recipient is fully responsible for any harm to themselves or third parties arising from their use, handling, or storage of Biological Materials. The recipient may not claim any rights against the Institute of Food Science and must compensate the institute for any damages or costs resulting from such incidents.
4. Intellectual Property: The transfer of Biological Materials does not include the authorization or transfer of intellectual property rights or ownership. The Institute of Food Science does not guarantee that their use will not infringe on the intellectual property or other rights of others. The applicant agrees to grant the institute the right to use any intellectual property derived from the use of Biological Materials for general business purposes, including transfer, identification, research, and services.
5. Compliance with Regulations: The applicant must comply with relevant laws and regulations regarding the use of Biological Materials, including transport, import/export, disposal, biosafety, and experimental procedures.
6. Publication Acknowledgment: In any publications (including patent applications) related to Biological Materials or their Modified Materials, the applicant must acknowledge that the materials were obtained from the Biological Resource Preservation and Research Center at the Institute of Food Science, including the source and catalog number as listed in the center's directory.
7. Prohibition on Re-transfer: Biological Materials may not be transferred to third parties in any form.
8. Prohibition on Human Trials: Biological Materials may not be used for human trials.
9. Operational Qualifications: The applicant understands that if the Institute of Food Science determines that the applicant lacks the necessary expertise, biosafety equipment, or operational capability, the institute reserves the right to refuse the transfer.
10. Limitation of Liability: The Institute of Food Science is committed to providing viable and uncontaminated Biological Materials. If the materials received are not viable or are contaminated, the applicant must notify the institute in writing within a specified period, and the institute will provide a replacement or other compensation (limited to the order amount for the Biological Materials). Aside from this, the institute makes no warranty regarding the safety, suitability, or accuracy of the Biological Materials or related information.
11. Special Terms: Any "special requirements" listed in individual Biological Materials catalogs or product descriptions are considered part of this agreement. In case of conflict, the "special requirements" take precedence.
12. Other Terms: None

財團法人 食品工業發展研究所
生物資源保存及研究中心

地址：新竹市食品路331號　網址：http://www.bcrc.firdi.org.tw
電話：03-5223191轉509或248　傳真：03-5224172或03-5224171

產品說明書

生資中心編號：	60038
細胞株名稱：	Hs68
組織來源：	Tissue：skin; foreskin; fibroblast; aspartoacylase deficiency; possible Canavan disease<br>Species：*Homo sapiens* (human)
冷凍管容量：	1 ml
濃度：	0.88 x 10^6 cells/ml
冷凍日期：	2021/03/16
繼代數：	P=19
存活率：	97.8%
冷凍批號：	Lot-02631
生長特性：	Adherent
形態：	Fibroblast
培養基 ：	Culture Medium：90% Dulbecco' s modified Eagle' s medium with 4 mM L-glutamine adjusted to contain 1.5 g/L sodium bicarbonate and 4.5 g/L glucose + 10% fetal bovine serum
培養條件：	37℃, 5% CO_2
冷凍培養基：	93% culture medium + 7% DMSO
培養基更換：	every 2 to 3 days
繼代培養：	吸除培養基，用PBS洗滌細胞1-2次後移去PBS，加入trypsin-EDTA溶液，放在37℃作用數分鐘後，輕拍培養瓶使細胞自瓶壁脫落，加入含血清之新鮮培養基，均勻混合後轉移至新的培養瓶中。
繼代稀釋比例：	1:4 to 1:8
污染測試：	negative for bacteria, fungi, and mycoplasma
